# SERS Tags for Biomedical Detection and Bioimaging

**DOI:** 10.7150/thno.66859

**Published:** 2022-01-24

**Authors:** Huiqiao Liu, Xia Gao, Chen Xu, Dingbin Liu

**Affiliations:** 1 College of Chemistry and Chemical Engineering, Xinyang Key Laboratory of Functional Nanomaterials for Bioanalysis, Xinyang Normal University, Xinyang 464000, China.; 2 State Key Laboratory of Food Nutrition and Safety, College of Food Science and Engineering, Tianjin University of Science and Technology, Tianjin 300457, China.; 3 Department of Colorectal Surgery, Tianjin Union Medical Center, Tianjin Institute of Coloproctology, Tianjin 300000, China.; 4 State Key Laboratory of Medicinal Chemical Biology, Research Center for Analytical Sciences, and Tianjin Key Laboratory of Molecular Recognition and Biosensing, College of Chemistry, Nankai University, Tianjin 300071, China.

**Keywords:** SERS tag, biomarkers, tumor, bioimaging, theranostics

## Abstract

Surface-enhanced Raman scattering (SERS) has emerged as a valuable technique for molecular identification. Due to the characteristics of high sensitivity, excellent signal specificity, and photobleaching resistance, SERS has been widely used in the fields of environmental monitoring, food safety, and disease diagnosis. By attaching the organic molecules to the surface of plasmonic nanoparticles, the obtained SERS tags show high-performance multiplexing capability for biosensing. The past decade has witnessed the progress of SERS tags for liquid biopsy, bioimaging, and theranostics applications. This review focuses on the advances of SERS tags in biomedical fields. We first introduce the building blocks of SERS tags, followed by the summarization of recent progress in SERS tags employed for detecting biomarkers, such as DNA, miRNA, and protein in biological fluids, as well as imaging from *in vitro* cell, bacteria, tissue to *in vivo* tumors. Further, we illustrate the appealing applications of SERS tags for delineating tumor margins and cancer diagnosis. In the end, perspectives of SERS tags projecting into the possible obstacles are deliberately proposed in future clinical translation.

## Introduction

Surface-enhanced Raman scattering (SERS) has gained ever-increasing attention since its discovery. As a technique for detecting molecules near the surface of plasmonic nanostructures, SERS has been employed recently in biological and medical applications 1-3. Compared with other methods for biomolecules detection, SERS presents the following advantages: providing unique “fingerprint” information of biomolecules, ultrahigh sensitivity for non-invasive single-molecule detection, resistance to photobleaching and photodegradation, multiple detection capabilities, and negligible interference of water, which make it proper for studying complex biological systems in the active state. Direct sensing of analytes represents the classical application of SERS. By attaching the analytes to a plasmonic SERS substrate, their inherent Raman scattering signals are significantly enhanced, which can be employed for quantitative analysis and structural identification 4. The remarkable Raman scattering enhancements of the analytes are mainly attributed to the high electromagnetic fields near the plasmonic substrates. Electromagnetic enhancement is associated with the interaction of plasmonic nanostructures with electromagnetic waves. When the collective oscillation frequency of free electrons in nanostructures is in resonance with the external electromagnetic radiation, the strength of the electric field confined on the surfaces of the nanostructures will be enhanced, which would strongly influence the polarizability of the near molecules. As a result, Raman signals of the molecules located on the surface of plasmonic nanostructures will be enhanced significantly 5.

Direct SERS analysis is compelling for identifying the analytes with large cross-sections. However, in the context of biological analysis, it is still a real challenge for directly detecting biomolecules with extremely low intrinsic Raman cross-sections. Indirect SERS analysis with SERS tags has been proposed and developed rapidly in recent years 6-8. Organic molecules with large Raman cross-sections are employed as Raman reporters and modified on the surface of plasmonic nanoparticles. After being protected by coating layers and functionalized with bioligands, these fabricated SERS tags with clear Raman characteristic peaks are employed for probing biomolecules. By using laser Raman spectrometry or SERS microscopy to record Raman signals, SERS tags are successfully applied for biomedical applications.

The use of SERS tags for bioanalysis has been widely investigated to detect analytes in biofluid samples 9-12. In this case, the specific Raman spectra of SERS tags indicate the presence of DNA, proteins, and other biomarkers. Raman intensity or frequency of these spectra is commonly used for quantifying or identifying targets. In addition, SERS imaging has gained extensive attention due to its versatility, non-invasive character, and negligible photobleaching. SERS tags-based imaging for isolated living tumor cells, post-mortem excised tumor tissues, and *in vivo* tumors has proven feasible 13-15. The invasive margin of the tumor xenograft can be delineated by intraoperative SERS imaging, which is beneficial for guiding tumor surgery 16-18. Moreover, SERS tags have been endowed with multiple roles by integrating imaging with other functions (such as photothermal therapy (PTT) and photodynamic therapy (PDT)) for simultaneous diagnosis and treatment 19-21. Therefore, SERS tags show great potentials in clinical applications.

In this review, we will focus on state-of-the-art applications in biomedical with SERS tags. Starting with the building blocks of SERS tags, we introduce the fabrication process and the design principle of SERS tags, followed by the topics in biomedical applications based on SERS tags. We first summarize the recent progress of biomarkers in biological fluids and cells detected by SERS tags. Subsequently, we move the focus to the application of SERS tags for biomedical imaging ranging from *in vitro* cellular imaging to *in vivo* tumor imaging. Further, the exciting applications of SERS tags in the clinic, including the delineation of tumor margins and the integration of diagnosis and therapy, are introduced. Finally, we provide perspectives on the possible obstacles of SERS tags employed in future clinical translation.

### Building blocks of SERS tags

**Scheme 1:**
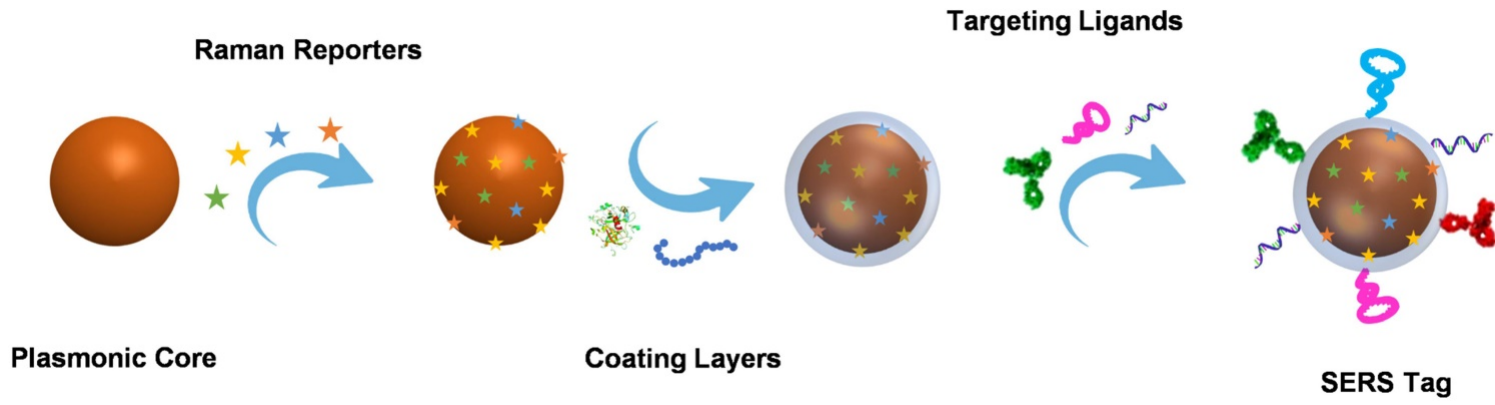
Building blocks and preparation process of a SERS tag.

As a signal output source for indirect detection, a SERS tag usually consists of a plasmonic nanoparticle core, a layer of Raman reporters, a protective coating shell outside the Raman reporters, and targeting ligands on the protective shell. Plasmonic nanoparticle core has the mission to enhance the Raman signals, whose chemical composition, size, and shape significantly affect the performance of SERS tags. The enhanced Raman signal of the reporters on the surface of the plasmonic nanoparticle may indirectly reflect the amounts of analytes when the SERS tags are employed for bioanalysis. Due to the complexity of biological samples, the structure that Raman reporters attached to the plasmonic core may become unstable; the protective layer appears to be essential. The outmost targeting ligands are needed to endow SERS tags with the ability to detect biomolecules selectively. The typical preparation process of a SERS tag is illustrated in **Scheme 1**.

In general, to better employ the SERS tag for biomedical applications, brightness is a critical factor that should be considered when designing a SERS tag. The brightness of the SERS tag is influenced by the effect of SERS enhancement factor, the number of Raman reporters, and the molecular cross-section. To enhance the brightness, there are several principles to follow. First, we can improve the SERS enhancement factor of the plasmonic nanoparticle cores. Compared to the typical ones, plasmonic cores bearing intense hot spots have come into notice with enhanced enhancement factors, such as dimers, aggregates, gap-embedded cores, and porous cores. In addition, by adjusting the Raman reporters, like choosing reporters with larger Raman cross-sections or increasing the effective number of reporter molecules, the brightness of SERS tags could also be improved. Moreover, in the past decades, eliminating background has become another fashion to improve the sensitivity of SERS tags by increasing their signal-to-background ratio (SBR). The SBR, defined as the level of the desired signal relative to the background signal, is the key element to realize the detection of low-abundance targets, especially in complicated samples. In this regard, different from the conventional nanotags that exhibit multiple bands in the fingerprint region (<1800 cm^-1^), Raman tags possess characteristic peaks in the so-called Raman-silent region (1800-2800 cm^-1^) have drawn the attention, where no signals can be detected for endogenous biomolecules, meaning zero background noise. To this end, molecules with chemical groups, such as alkyne, azide, nitrile, deuterium, and metal-carbonyl have been used as Raman reporters to fabricate background-free SERS tags for bioanalysis and bioimaging.

Additionally, to obtain reliable results for biomedical analysis, the uniformity and stability of SERS tags are another two important issues that should be considered carefully. By employing liquid phases synthesis or top-down lithography to fabricate uniform-sized plasmonic cores, as well as getting helping hands from protective shells, such as polymer, silica, biomolecules and metal-organic frameworks (MOFs), the obtained SERS tags tend to be highly uniform and stable. Furthermore, the factor of penetration depth should not be overlooked when designing SERS tags, especially for *in vivo* bioanalysis or bioimaging. In recent years, SERS tags for *in vivo* applications have attracted much attention and become the subject of detailed reviews 1, 22. Considering the low background signals from tissues in the near-infrared (NIR) region, the so-called NIR-SERS tags have been widely developed for biomolecular imaging. On the one hand, it is preferred to design the SERS substrates whose SPRs appeared in the NIR region. On the other hand, the maximum Raman cross-section of the reporter molecules should be resonant with the excitation wavelength of NIR lasers (e. g., 785 nm and 1280 nm laser). To this end, the obtained NIR-SERS tags would exhibit high penetration depth and sensitivity under the irradiation of NIR lasers for *in vivo* analysis 23, 24.

### Plasmonic nanoparticle cores

**Figure 1:**
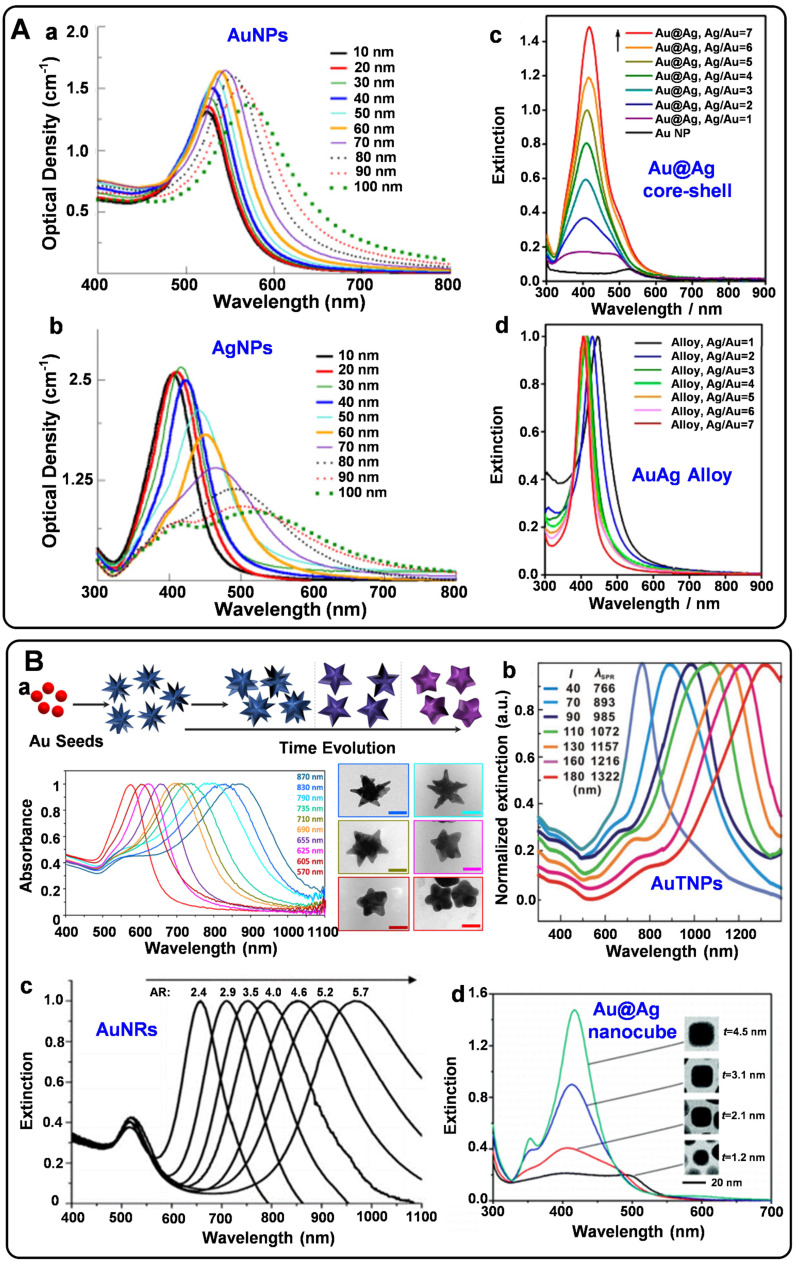
A) Optical properties of spherical plasmonic nanoparticles. (a) Extinction spectra of AuNPs with diameters ranging from 10-100 nm. (b) Extinction spectra of AgNPs with diameters ranging from 10-100 nm. (c) Extinction spectra of Au@Ag core-shell nanospheres and (d) extinction spectra of AuAg alloy nanospheres with different Ag/Au ratios. Adapted with permission from Ref. 12, 28, copyrights 2017 Elsevier, 2014 American Chemical Society. B) Optical properties of non-spherical plasmonic nanoparticles. (a) The schematic illustration of the synthesis of AuNSs and the UV-vis spectra of different AuNSs during the overgrowing process. (b) Normalized UV-vis-NIR extinction spectra of the AuTNPs with different average edge lengths. (c) Extinction spectra of AuNRs with different aspect ratios. (d) Extinction spectra of Au@Ag core-shell nanocubes with different thicknesses of Ag shells. Adapted with permission from Ref. 29, 30, 32, 33, copyrights 2018 American Chemical Society, 2009 Wiley-VCH, 2010 American Chemical Society, 2014 Royal Society of Chemistry.

Spherical plasmonic nanoparticles, such as gold silver nanospheres (AuNPs and AgNPs), Au@Ag core-shell nanospheres, and AuAg alloys, are the most widely used plasmonic substrates for fabricating SERS tags. According to the electromagnetic enhancement mechanism, when the surface plasmons of metal NPs are excited by proper laser radiation, the localized electromagnetic fields will generate on the NP surfaces. Since the absorption bands of spherical AuNPs, AgNPs, Au@Ag core-shell nanoparticles, and AuAg alloys appear in the visible region (**Figure 1**A), they are conveniently detected by the readily available 532 nm and 633 nm lasers. AuNPs are usually prepared by reducing HAuCl_4_ at high temperatures or seed-mediated methods 25, 26. The as-prepared AuNPs with sizes ranging from 10 to 100 nm display well water-solubility and dispersibility. As the sizes increase, the extinction peaks show red-shift tendencies. AgNPs have been employed for Raman enhancement since they were first synthesized by reducing AgNO_3_ with sodium citrate in boiling water in 1982 27. It is hard to regulate the sizes of AgNPs by adjusting the amounts of reactants. Although AgNPs have excellent SERS enhancement performance, the surfaces of AgNPs are easily oxidized to form the SERS passivation layers. Fabricating AgAu alloys nanoparticles is an effective strategy to protect AgNPs from oxidation and fully realize their Raman enhancement properties 28. In addition, biocompatibility and stability of the plasmonic nanospheres are two essential parameters for engineering SERS tags. Compared with AgNPs, AuNPs own good biocompatibility and long-time stability, which are more feasible for biological applications.

SERS tags should be highly bright for the sensitive detection of biomolecules. For this purpose, some non-spherical plasmonic nanoparticles have been developed for engineering SERS tags in recent years. Typically, plasmonic substrates with nano-gaps, tips, and edges usually present excellent plasmonic near-field enhancements, providing the enhancement factors up to the order of 10^9^-10^10^ 6. Non-spherical plasmonic nanoparticles such as gold nanostars (AuNSs), gold nanorods (AuNRs), gold triangle nanoplates (AuTNPs), and gold nanocubes are commonly synthesized by multistep methods. By the seed-mediated method, AuNSs can be obtained in the presence of reducing agents, stabilizers, and shape inducers 29. The Raman enhancement properties of nanostars are greatly influenced by their spike number, length, and sharpness. When adding the gold nanoparticle seeds into the growth solution, the seed crystals are grown into AuNRs with definite aspect ratios under the regulation of surfactants 30. The Raman enhancement factor provided by AuNRs depends on their sizes, ratios of diameter to length, and radius of curvature. Besides, by selectively protecting the surface of the seed crystals, the growth rates of different facets can be precisely controlled. Thus, plasmonic nanoparticles with edges and corners are synthesized 31-33. When employed for engineering SERS tags, these non-spherical plasmonic nanomaterials present powerful local electromagnetic fields, which is beneficial to improve the brightness of SERS tags. Moreover, the extinction peaks of most non-spherical plasmonic nanoparticles can be tunable from ultra violet-visible (UV-vis) to NIR (**Figure 1**B). These plasmonic substrates may exhibit strong SERS responses when excited at the wavelength of 785 nm, endowing them the ability for *in vivo* applications.

Nano aggregates with huge hot spots can also be employed to design SERS tags. The electromagnetic intensity in hot spots of aggregates is over 1000 times stronger than that of the single spherical plasmonic nanoparticles, providing the main effect for Raman enhancement 34. The fabrication of aggregates is mainly divided into bottom-up assembly and top-down lithography. Salts or dyes in the solution may induce the aggregation of single nanoparticles, forming clusters with a large number of nanogaps. It is worth noting that although the aggregated plasmonic NPs present high SERS responses, the degrees of the aggregation should be well weighed when they are employed as SERS tags. Too large aggregated SERS tags may cause slow kinetics and nonspecific adsorption. The random distribution of hot spots in small aggregates would result in a nonlinear dependence of the SERS signal on tag quantity 35. Sculpting single plasmonic nanoparticles is one of the most used methods to construct controllable hot spots in SERS tags. For example, through the processes that prepare AuAg or AuCu alloys first and followed with the selective dissolution of Ag or Cu elements, nanoparticles with numerous nanogaps can be obtained 36, 37. Moreover, the size and number of the gaps can be regulated by the amounts of sacrificial agents and etching times.

### Raman reporters

The molecules selected as Raman reporters should have the following characteristics: (1) own large Raman scattering cross-sections to generate intense Raman signals, (2) possess the ability for linking to the surface of plasmonic nanoparticles through chemical bonds or physical interactions, (3) have as few as Raman peaks to reduce the spectral overlap in the multiplexing experiments, (4) show high stability under laser irradiation. Typically, the aromatic molecules containing nitrogen or sulfur elements are the most commonly used Raman reporters due to their high affinity to Au and Ag. A large Raman reporter library with different Raman codes has been built directly using aromatic compounds 26. The SERS tags engineered by these Raman reporters are extensively applied for *in vitro* bioanalysis. For example, the Raman reporter of 4-mercaptobenzoic acid (4-MBA) assembled on the surface of Ag nanocube was used as SERS tag for sensitive detection of oral cancer DNA 38. SERS tags prepared by modifying AuAgNPs with a Raman reporter molecule of 4-mercaptobenzonitrile (4-MBN) were used for multiple cancer-associated miRNAs 39. Besides, by conjugating nanoboxes with the Raman reporters of 5,5-dithiobis (2-nitrobenzoic acid) (DTNB), 4-MBA, 2,3,5,6-tetrafluoro-4-mercaptobenzoic acid, and 2‐mercapto‐4‐methyl‐5‐thiazoleacetic acid, Li *et al*. fabricated four SERS tags with different Raman spectra, which were used for multiplexed cytokine analysis 10. Some typical Raman reporters are illustrated in **Table 1**.

**Figure 2:**
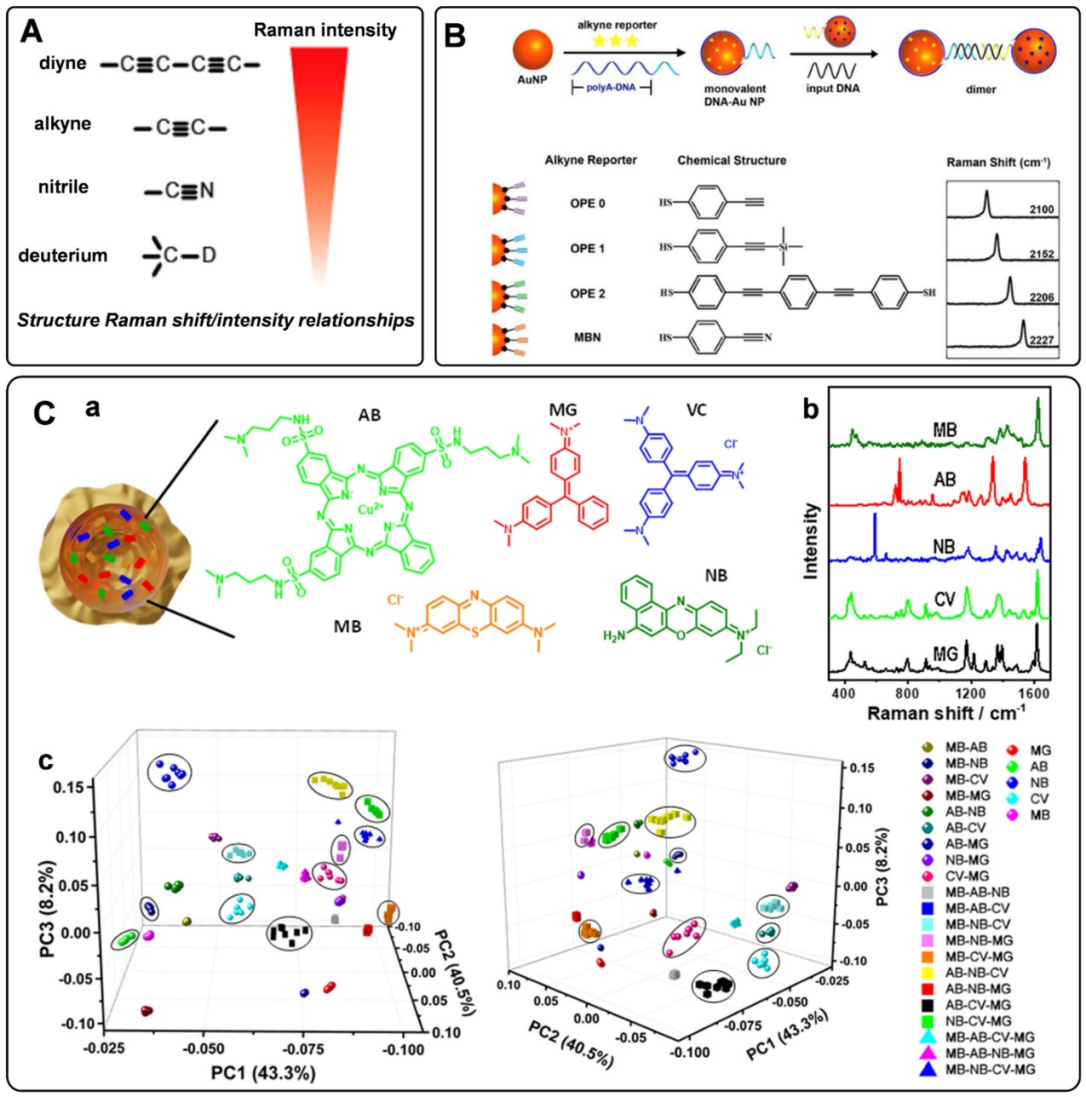
A) The relative intensity of bioorthogonal Raman reporters with typical groups. Adapted with permission from Ref. 52, copyright 2012 American Chemical Society. B) The design protocol of click SERS by nanoparticle dimerization, as well as the chemical structures and Raman shifts of the used four triple bonds-based Raman reporters. Adapted with permission from Ref. 54, copyright 2018 American Chemical Society. C) (a) and (b) Schematic representation of a plasmonic nanocapsule encoded with five different Raman reporters and their representative SERS spectra. (c) 3D PCA score plots for the first three PCs from 26 SERS tags obtained by the combination of five different Raman reporters. Adapted with permission from Ref. 58, copyright 2020 American Chemical Society.

When designing the SERS tags for *in vivo* applications, it should be noted that the endogenous biological components generate Raman shifts in the fingerprint region. To avoid the Raman frequency overlapping of SERS tags and endogenous biological molecules, a class of bioorthogonal Raman reporters has been reported for fabricating SERS tags. Bioorthogonal Raman reporters present strong Raman signals in the biologically Raman-silent region. Alkyne-tagged SERS probes are commonly used Raman reporters for imaging bioactive molecules 49. When the triple bonds are conjugated with different organic skeletons, the Raman shifts in the silence region show a clear difference, dramatically expanding the category of SERS tags encoded by alkyne 50. By rational engineering of the conjugation length, bond-selective isotope doping, and end-capping substitution of polyynes, Min's group achieved 20 distinct Raman reporters with frequencies in the Raman-silent region 51. Analogously, nitrile, azide, and deuterium are promising functionalities for bioorthogonal Raman reporters due to their powerful Raman peaks in the cellular silence region. As reported by Yamakoshi *et al*., the relative intensities of the bioorthogonal Raman reporters are shown in **Figure 2**A 52. Raman characteristic peaks of diyne and alkyne exhibited the highest signals, followed by nitrile and deuterium. Although the Raman intensity of nitrile is lower than that of alkyne, it is still widely used as a Raman tag. Our group has employed nitrile- and alkyne- terminated molecules to fabricate SERS tags and furtherly realized the *in situ* detection of multiple microRNAs in the cell 53. To enhance the coding ability of Raman reporters, Zeng *et al*. proposed a “click SERS” strategy for multiplexing liquid biopsy and accurate cellular imaging 54. As illustrated in **Figure 2**B, they used four different triple bond-containing dyes to modify AuNPs. The SERS active nanoparticles spliced together with the presence of targets. Based on the number and frequency position of the delivered Raman spectra, ten kinds of biomarkers could be analyzed simultaneously. More recently, two metal carbonyl (metal-CO) labels have been reported as novel interference-free labels by Lin *et al*. 55. The characteristic peaks of Os_3_(CO)_9_(μ-CO)(μ_3_-S) and Re_2_(CO)_8_(μ-SC_6_H_5_) were located at 2025 and 2113 cm^-1^, different from that of biological fluids. Moreover, both molecules possessed thiolate groups, which were beneficial to the interaction of metal-CO with plasmonic nanoparticles.

Given that the Raman reporters' sensitivity is proportional to their scattering cross-sections, some fluorophores, such as cyanine dyes, are often used as parent structures for modification. When the Raman reporter displays an electronic transition in resonance with the excitation laser wavelength, the Raman intensity will be further enhanced, which is defined as surface-enhanced resonance Raman scattering (SERRS) 7. Moreover, SERRS tags encoded with NIR Raman reporters show great potential for *in vivo* detection owing to their super sensitivity. The weak interaction force between NIR dyes and plasmonic nanoparticles makes it hard to modify Raman reporters densely and stably on the metal surfaces. Encapsulating these dyes into a shell or cavity is an effective strategy to prepare stable and sensitive SERS tags with NIR Raman reporters. By using lipid bilayer to encapsulate the NIR Raman reporters on the surface of Au nanoparticles through hydrophobic interactions, thiol group-free NIR Raman reporters could be modified on the surface of Au nanoparticles 56. Besides, IR780 perchlorate or IR792 perchlorate can be wrapped by the silica coating layer to produce the SERS tags 57. The above methods endow SERS tags with super sensitivity and stability when used for biomedical detection.

To further expand the library of Raman reporters, Rodal-Celeira *et al.* proposed a universal method to fabricate SERS tags by encapsulating Raman reporter molecules into metal nanoshells 58. With this strategy, five different group-free Raman reporters could be trapped by plasmonic nanocapsules (**Figures 2**C, a-b). There would be many different possible codes when vesting each Raman reporter binary code according to its presence or absence in the labeled SERS tag. These SERS tags would produce high amounts of data when used for multi targets detection simultaneous. To better illustrate the degree of correlation or covariance among data, some appropriate data analysis methods have been developed in recent years. Principal component analysis (PCA) is one of the most used methods for reducing the data dimensionality without losing information related to the variability of the sample 59. With this method, the main components in the combination of five different SERS tags prepared by trapping different Raman reporters into plasmonic nanocapsules could be identified (**Figure 2**C, c). Besides, based on the model predicted from data features, multiple linear regression has also been used to decompose the SERS spectra of multiple SERS tags to component SERS tag spectra 60.

### Protective coating shells

The noble nanostructures will show characteristic Raman peaks after being modified with Raman reporters. Nonetheless, they cannot be used directly for biomolecular detecting or imaging. The Raman reporters would dissociate from noble nano substrates in complex biochemical environments, resulting in the fluctuation of Raman signals. Surface coating is necessary to protect the probes from the interface of physiological fluids, thus making the SERS tags are reliable and efficient for advanced applications. Besides, increasing biocompatibility and lowering biotoxicity are also beneficial effects through the surface coating process. In recent years, various surface coating materials, including silica, polymers, biomolecules, and metals, have been adopted for different applications of SERS tags.

**Figure 3:**
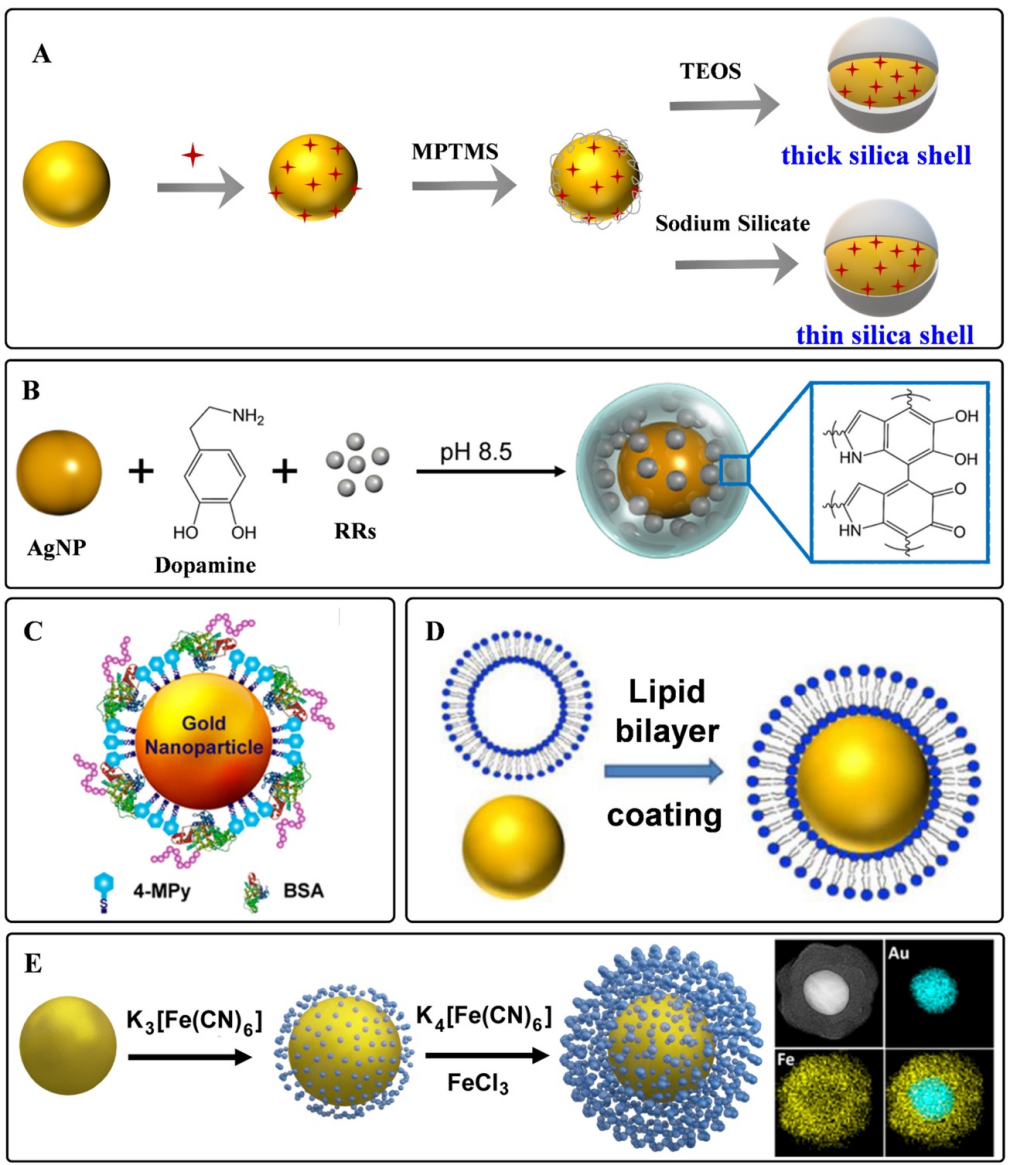
Representative protective coating shells: A) Schematic diagram of silica as protective to coated on the surface of plasmonic nanoparticles. B) Schematic one-step synthesis of SERS tags with PDA as protective shells. Adapted with permission from Ref. 65, copyright 2018 Royal Society of Chemistry. C) Schematic diagram of BSA on the surface of plasmonic nanoparticles. Adapted with permission from Ref. 67, copyright 2019 American Chemical Society. D) Lipid bilayer-assisted synthesis of SERS tags. Adapted with permission from Ref. 70, copyright 2018 American Chemical Society. E) The preparation process of Au@PB. Adapted with permission from Ref. 74, copyright 2017 American Chemical Society.

Silica is one of the materials used earlier as a protective coating layer of SERS tags, which has been commercialized in recent years. The silica sources are mainly 3-aminopropyltrimethoxy silane (APTMS), 3-mercaptopropyl trimethoxy silane (MPTMS), sodium silicate, and tetraethyl orthosilicate (TEOS), etc. Monolayered MPTMS or APTMS molecules can attach to the surface of noble nanostructures by forming metal-S or metal-N bonds. Sodium silicate or TEOS are employed to adjust the thickness of silica coating layers (**Figure 3**A) 56, 61. Sodium silicate is the optimized silica source for engineering the SERS tags with ultrathin silica layers, and the thick silica coating layers are always formed through the hydrolysis of TEOS.

Polymers have the characteristic of biocompatibility, stability, and are easy to get, which have been widely used to construct coating layers of SERS tags. Mercapto functionalized polyethylene glycol (SH-PEG) is one of the most commonly used polymers to protect noble nanoparticles from aggregating. SH-PEGs with different molecular weights have been proved to increase the stability and retain the biocompatibility of SERS tags 45. Meanwhile, they can also provide chemical end groups, rendering the SERS tags amenable to surface functionalization. Polyethylene pyrrolidone 62, polystyrene 63, and chitosan 64 have also been used for stabilizing nanoparticles. Our group proposed a universal method for the one-pot synthesis of SERS tags with the assistance of polydopamine (PDA) 65. As illustrated in **Figure 3**B, Raman reporters can be attached to nanoparticle surfaces during dopamine polymerization. With this strategy, 82 background-free Raman reporters were wrapped successfully by PDA to prepare SERS tags. Moreover, the exposed dopamine quinone could couple with targeting ligands by Michael addition.

Biomolecules are also employed as protective shells to increase the biocompatibility of SERS tags. Among them, bovine serum albumin (BSA) is widely used to protect the nanoparticle cores 66, 67. BSA can act as coating layers on the surface of nanoparticles by physical absorption (**Figure 3**C). When used for a long-time *in vivo* monitoring, it should be noted that the weak interactions between plasmonic nanoparticles and BSA may lead to aggregation of the BSA-protected SERS tags. Phospholipids are also reported for encapsulating nanoparticles owing to their inherent biocompatibility self-assembly ability (**Figure 3**D). The phospholipid coating layer endows the SERS tag with good biocompatibility and colloidal stability and the ability to target biomolecules 68, 69. Besides, the liposomes can also serve as flexible scaffolds for assembling small nanoparticles outside liposome layers. By taking lipid bilayer as cross-linker, Mei *et al*. fabricated waxberry-like core-satellite nanoparticles by *in situ* growth of small AuNPs on phospholipid bilayer-coated gold cores 70. This unique structure exhibited extraordinary SERS ability owing to the numerous hot spots at the junctions.

As a kind of hybrid organic-inorganic supermolecule materials, MOFs exhibit excellent performance in sensing, catalysis, and energy storage. Moreover, they have been proved to enhance Raman signals owning to the charge transfers between MOFs and probe molecules 71. When employing MOFs to encapsulate plasmonic nanoparticles, the packaged structures show enhanced stability and sensitivity. Plasmonic nanoparticles with different shapes could be encapsulated by zeolitic imidazolate framework-8 (ZIF-8) 72, 73. By meticulous designation of a thick ZIF-8 shell on the surface of AuNRs, Lafuente et al. fabricated a quantitative and sensitive SERS platform 73. The MOF shells assumed the role of filtration to prevent interferences from closing to the surface of AuNRs, strengthening the identification capability of the sensors. In addition, Prussian blue (PB) and analogs have also been investigated to be coating layers of SERS tags (**Figure 3**E). These coordination shells are formed with CN-bridged cubic structures. The number of C≡N bonds in the PB coating shell is far more than that in the traditional organic molecules protective shell, which is beneficial for enhancing the sensitivity of SERS tags. Moreover, the C≡N groups present a specific, strong, and stable SERS emission in the Raman-silent region, making it promising for *in vivo* imaging with none-background signals 21, 74.

### Targeting ligands

For tracking specific targets, the SERS tags should be further functionalized with bio-recognition molecules, such as oligonucleotides, peptides, antibodies, and aptamers. Sulfhydryl group-containing biomolecules can be modified on the surface of Au or Ag core via Au-S or Ag-S bonds. In this case, competition will occur between the targeting ligands and protective molecules fastened by metal-S bonds. Without replacing or breaking the protective layers, targeting ligand molecules can be linked outside of them by chemical reactions. Peptides and antibodies can be linked to plasmonic nanoparticles via the reaction of carboxylic acid groups and amine groups in the presence of 1-ethyl-3-(3-dimethylaminopropyl) carbodiimide and N-hydroxy succinimides. In addition, biotin-modified antibodies or aptamers can be linked to the streptavidin-tagged nanoparticles by the strong affinity between biotin and streptavidin 57, 75. Peptides terminated with cysteine can also be conjugated to the BSA layer by forming S-S bonds 67. Alternatively, electrostatic interactions have also been used for antibodies with negative electronic with poly-L-lysine modified plasmonic cores 74.

### Detection of Biomarkers with SERS tags

*In vitro* diagnosis is vital for monitoring various types of cancer at the preliminary stages. Biomarkers such as DNAs, miRNAs, proteins, and other biomolecules have been investigated for early cancer diagnosis and prognosis. Precise monitoring of these biomarkers will be highly useful for adopting preventive measures 76. The specificity and sensitivity of the SERS tag make it possible for accurate detection of the physiologically relevant analytes in complex biological fluids. For detecting the biomarkers, the SERS tags are labeled with corresponding recognition molecules (such as antibodies and aptamers) to allow specific sensing of targets with Raman signals. With this concept, multitarget detection platforms based on the SERS tags have also been developed with superior multiplexing capability and high sensitivity. The advances in detecting disease-related biomarkers with SERS tags will be discussed in this part.

### Detection of DNAs with SERS tags

**Figure 4:**
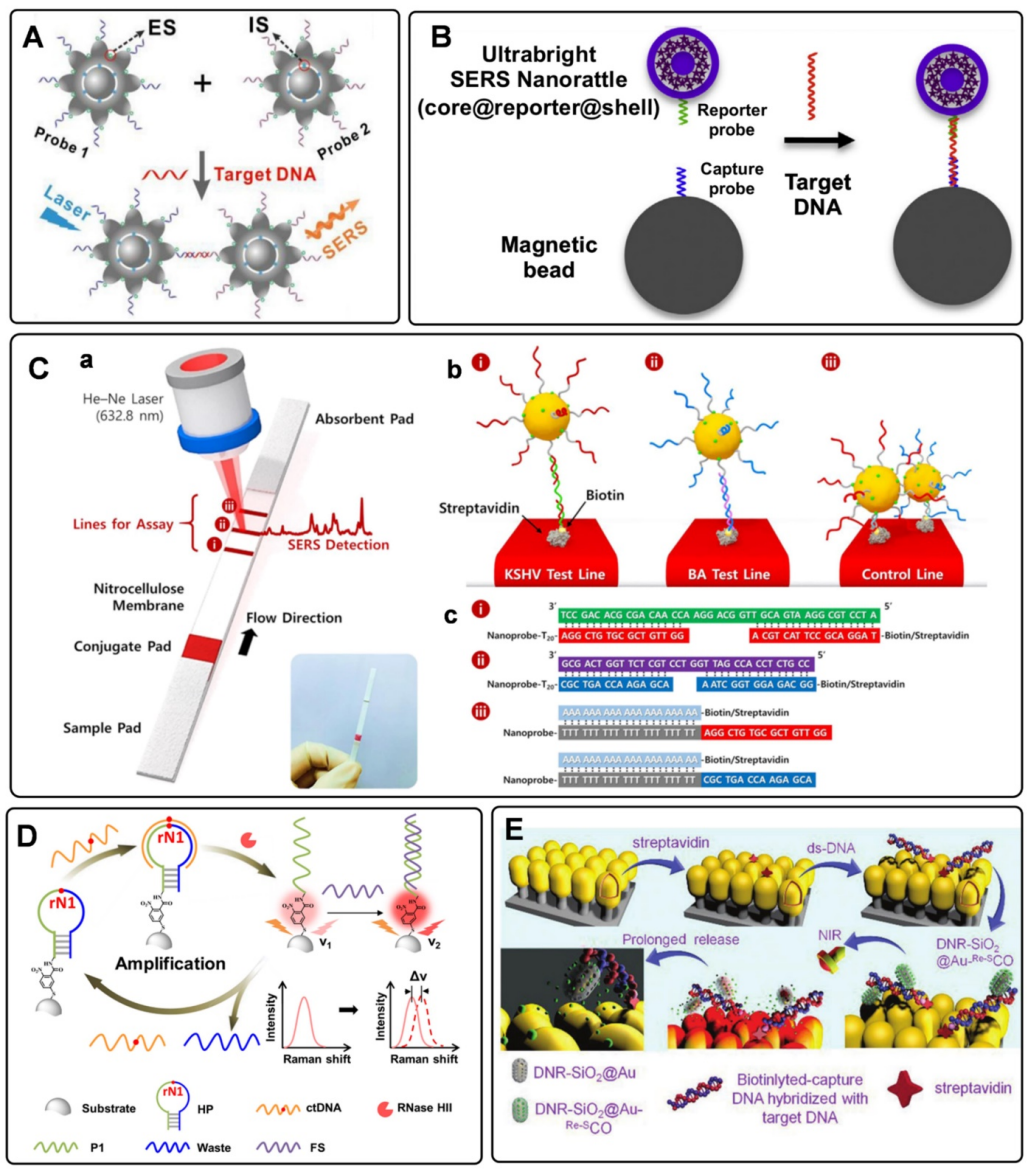
A) Schematic diagram of the sandwich-type assay for detection of nucleic acids. Adapted with permission from Ref. 83, copyright 2020 Elsevier. B) The schematic illustration of DNA detection with sandwich hybridization of MB, target sequence, and ultrabright SERS tags. Adapted with permission from Ref. 84, copyright 2016 Elsevier. C) Schematic illustration of the lateral flow strip biosensor for the simultaneous detection of two nucleic acids. (a) The strip is composed of two test lines and one control line. (b) SERS tags were captured by the specific region. (c) Corresponding DNA hybridizations for test and control lines. Adapted with permission from Ref. 87, copyright 2017 American Chemical Society. D) Schematic diagram of an RNase HII-mediated signal amplification platform for DNA detection based on frequency shift. Adapted with permission from Ref. 92, copyright 2019 American Chemical Society. E) The mechanism scheme of signal amplification by increasing the turnover rate of the SERS signal. Adapted with permission from Ref. 55, copyright 2019 Royal Society of Chemistry.

Circulating tumor DNAs are produced by tumor cells during necrosis or apoptosis. It provides direct information on the genetic alterations and mutations in the solid tumor. Rapid and accurate detection of DNA has become a powerful tool for early cancer diagnosis 77, 78. Since Mirkin *et al*. proposed the sandwich assay for DNA detection with SERS tags 79, numerous works have been reported based on this principle. Generally, SERS tags are functionalized with partial sequences complementary to target DNAs. Meanwhile, solid substances are immobilized with the other complementary sequences to capture targets. The Raman intensities of these SERS tags indirectly reflect the amount of DNA targets, thus developing analytical methods for DNA based on SERS tags. The DNA detection sensitivity depends on the brightness of SERS tags. Numerous efforts have been made to fabricate hot spots in SERS tags by controlling plasmonic structures with nanogaps 80-82. Wang *et al*. developed a fractal SERS nanoprobe for the ultrasensitive detection of DNA 83. As illustrated in **Figure 4**A, the SERS tags were fabricated by an Au core and a stellate outer shell. There were numerous hollow gaps between the nucleus and outer shell in this structure, which was beneficial for loading Raman reporters in the gaps as internal standards (IS). Meanwhile, the outer shell provides distinct SERS enhancement for reproducible quantitative measurements of DNA.

To accurately detect the trace amount of specific DNA in biological fluids with SERS tags, preconcentration with a magnetic bead (MB) is an effective strategy. As shown in **Figure 4**B, sandwich-like structures are formed in the homogeneous solution with the components of the capture DNA probe-immobilized MB, the reporter probe-tied SERS tags, and the specific DNA targets. After that, these dispersed combinations gather together under the effect of an external magnetic field. As a result, the detectable Raman signals can be used for DNA analysis 84. Besides, magnetic particles coated with nanostructured plasmonic shells have also been fabricated. This unique structure integrates magnetic separation and SERS detection, showing excellent performance for DNA analysis 85.

SERS-based lateral flow strips have also been used for the *in vitro* detection of biomarkers. The integration of the conventional chromatographic separation and SERS tag readout makes the SERS-based lateral flow strip convenient for accurate DNA analysis. Fu *et al*. proposed a SERS-based lateral flow assay biosensor for DNA targets related to HIV-1 detection 86. In their protocol, the SERS tag packaged by Raman reporter and AuNPs was employed for probing the DNA target in a lateral flow strip. The Raman intensity of the SERS tag on the test line was used for quantitative analysis. This work provides new insight into the early diagnosis of HIV-1 disease. In addition, they further attempted to detect multiplex DNA biomarkers with the developed SERS-based lateral flow assay 87. As shown in **Figure 4**C, a, the strip with two test lines and one control line was designed for dual targets detection. Two types of SERS tags were captured by the specific region based on the nucleic acid engineering (**Figure 4**C, b and c). By analyzing the characteristic peak intensities of SERS tags on two test lines, two different kinds of DNA targets can be detected simultaneously. To further increase the multiplex detection capacities, a 2×3 microarray has been fabricated by Zhang *et al*. as the test zones on one nitrocellulose (NC) membrane 88. With this strategy, eleven DNA targets related to respiratory tract infections can be simultaneously detected with two Raman reporters encoded SERS tags.

Researchers have also proposed some novel platforms to improve the detection sensitivity of DNA by combining amplification strategies with SERS tags. Polymerase chain reaction (PCR) is a traditional technology for nucleic acid detection. The values of cycle threshold in amplification curves are always used for quantification. As a selectable method, the PCR products of fluorescence tag-labeled primers can also be measured by SERS techniques 89. It should be noted that the PCR products always present to be double-strand DNA, making it challenging to hybridize with functionalized SERS tags. To address this issue, Macdonald *et al*. proposed an asymmetric PCR-SERS method, by which single-strand DNA amplicons were synthesized and could be directly recognized by SERS tags 90. Other amplified techniques such as enzyme-boosted cascade reactions and heating assistant strategies have also been proved to improve the sensitivity of SERS-based DNA detection 38, 91. Zhang *et al*. designed a hairpin DNA-rN1-DNA probe for recognizing and hybridizing with ctDNA 92. As illustrated in **Figure 4**D, the DNA-rN1-DNA/ctDNA hybrid can be dissociated by RNase HII, leaving P1 stand on the surface of plasmonic nanoparticles and releasing ctDNA for the next cycle. Before and after the P1 sequence binding with a foreign sequence, the frequency shifts of the Raman reporter will change, which can be used for quantification. Besides, a signal amplification method that enlarges Raman reporters by a photo trigger has been reported 55. As illustrated in **Figure 4**E, the target DNA sequence was first captured by the biotinylated DNA. Then the formed double-strand DNAs were tied to a SERS substrate via biotin-streptavidin interaction. The complexes of daunorubicin and SERS tags were finally immobilized on the substrate by specifically chelating ds-DNA. When irradiating these SERS tags with an 808 nm laser, Raman reporters would escape from the mesoporous silica shell and then sprinkle on the Au nanopillar substrate. The high turnover rate of the Raman reporter will enhance Raman signals to a certain degree. The signal fluctuation can be corrected with IS embedded in the substrate, thus realizing the reliable quantitative SERS analysis of DNAs.

### Detection of miRNAs with SERS tags

Recently, miRNAs have emerged as a novel kind of biomarker since their aberrant expressions are associated with a variety of diseases, such as cancers, genetic disorders, and neurological 93. The inherent characteristics such as short lengths, highly-homologous sequences, vulnerable degradability, and low expression levels make miRNAs hard to be detected accurately. Recently, two main strategies, including nucleic acid engineering and target-induced SERS tags aggregation, have been adopted for miRNAs detection.

Different from the traditional detection module of one target to one probe, the miRNA detection platform with nucleic acid engineering presents high sensitivity with one target to multi-probes. Nucleic acid engineering can produce many cyclic products from a single target by rationally-designed nucleic acid sequences. Based on this concept, nuclease-assisted amplification methods have been adopted to fabricate miRNAs assays 94, 95. Briefly, DNA in the hybrid duplex chain of DNA and miRNA will hydrolyze in the presence of exonuclease or duplex-specific nuclease. After being treated with these enzymes, miRNAs will be released from the double strands for cycling. Highly sensitive miRNA assays would be built by combining the amplification strategy with SERS tags, in which Raman intensities of SERS tags were used for miRNA quantification. Rolling circle amplification (RCA) reaction is a commonly used strategy for DNA amplification. Combining RCA strategy with the functionalized chip and locked nucleic acid (LNA) probes, Zhu *et al*. reported a 3D organic nanoclusters SERS platform for miRNAs detection 96. The well-designed double-strand DNA immobilized on the chip would dissociate in the presence of miRNAs, leaving the single-strand DNAs as primers for RCA. By virtue of the interaction of biotin-streptavidin, the RCA products were hybridized with SERS tags to produce nanoclusters. These 3D organic-nanoclusters showed excellent Raman enhancement performance. Meanwhile, their Raman spectra were employed for the identification and quantitation of miRNAs.

**Figure 5:**
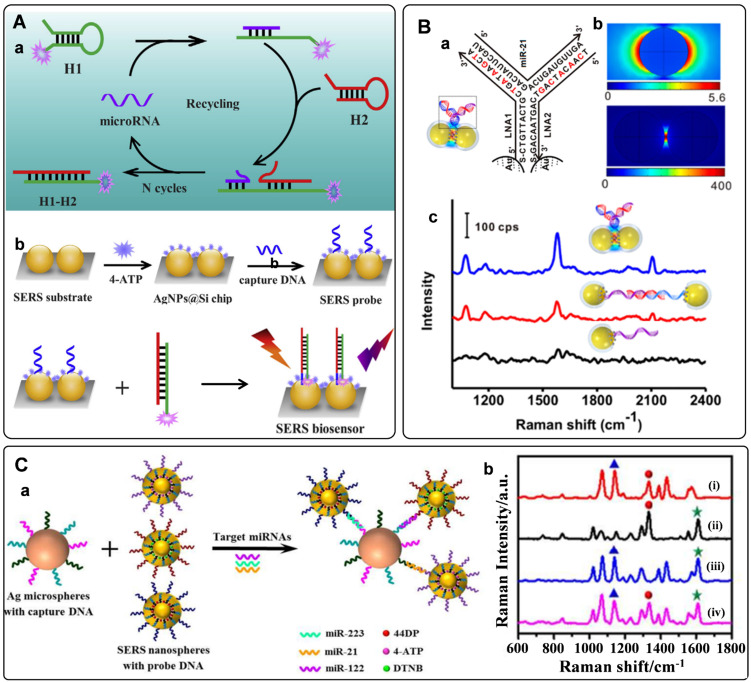
A) (a) Schematic of CHA amplification and (b) the SERS platform for quantitative detection of miRNA. Adapted with permission from Ref. 42, copyright 2019 Elsevier. B) Target-triggered SERS tag aggregation for miRNAs detection. (a) LNA sequences and miRNA-21 as hybridized in the Y-shaped dimers. (b) the calculated electromagnetic fields distribution of AuNPs individual and dimer. (c) Raman spectra of individuals, linear dimers, and Y-shaped dimers in the presents of target miRNA. Adapted with permission from Ref. 53, copyright 2017 American Chemical Society. C) (a) Schematic illustration of the multiplex SERS assay for multi-target miRNAs detection. (b) SERS spectra of the nanoprobes obtained in the presence of multiple miRNAs. Adapted with permission from Ref. 108, copyright 2017 American Chemical Society.

Owing to their enzyme-free and isothermal DNA reaction characteristics, strategies of catalytic hairpin assembly (CHA) amplification and hybridization chain reaction (HCR) have also been integrated with SERS tags for miRNAs detection 97-100. The process of CHA amplification is as follows: miRNA target first triggers the hybridization of hairpin structured H1 and H2. After that, the target will be released for the next cycle. The above processes promote the generation of dye-labeled DNA complexes, which would produce strong Raman signals when captured by SERS substrate (**Figure 5**A). With this strategy, the limit of detection for miRNAs can be as low as to fM level 42. Our group has proposed an *in situ* assembly strategy for single miRNA detection 101. The SERS tags were assembled into large particles via HCR assistance. The generated aggregations exhibited numerous hot spots to improve the detection sensitivity significantly.

Electromagnetic hot spots play the dominant role in Raman enhancement. Building hot spots in the SERS platform is an effective strategy to improve sensing sensitivity. In recent years, *in situ* fabrication of hot spots has been extensively studied for miRNAs detection 53, 102-105. Generally, plasmonic nanoparticles and the well-designed DNA are employed as assembly units, which can selectively turn on the SERS signal in the presence of miRNA target. Xu *et al*. presented the detection of miRNAs by side-by-side self-assembly of AuNRs dimers 104. Two single-strand DNA were firstly bonded to a pair of AuNRs. Then the twisted side-by-side dimers are formed by selective recognition of specific miRNAs sequences. The dyes labeled on the DNA would be located in the hot spot of AuNRs dimer, thus providing enhanced Raman spectroscopy. Further minimizing the interparticle distance will strengthen plasmonic coupling in the nanogaps. Based on this conception, our group designed a Y-shape duplex by LNA sequences hybridizing with the target miRNA (**Figure 5**B). The LNA modified SERS tags turned to be dimer in the presence of target miRNA. Compared with the linear dimers and individuals, a significant Raman enhancement was delivered by this Y-shaped construction 53. Furtherly, the miRNAs in cancer cells could be precisely monitored with this assay. With a similar strategy, the microRNA in exosomes were also detected with high sensitivities 105.

The occurrence and progression of disease often accompany abnormal expressions of multiple miRNAs. The simultaneous quantification of multiple miRNAs in biological samples holds great potentials for the early diagnosis of cancers 106. The characteristics of SERS tags, including ultrasensitivity, fingerprint, and narrow peaks, make them ideal probes for multiplex detection. Recently, numerous efforts have been made for multiple miRNAs detections with SERS tags 39, 107-109. A typical model for the detection of miRNA is a multiplex SERS-based sandwich hybridization. SERS tags encoded by Raman reporters are functionalized with different probe DNA sequences. Target miRNAs can be captured by one substrate and then hybridized with corresponding SERS tags, forming multiple sandwich complexes (**Figure 5**C, a). The types and amounts of miRNAs can be reflected by the Raman shifts and intensities of the complexes. With this strategy, the key for multi-target detection is preparing multiple SERS tags with non-overlapped Raman characteristic peaks. Zhou *et al*. fabricated three different SERS tags encoded by DTNB, 4-aminothiophenol, and 4, 4'-dipyridyl, respectively 108. The prominent peaks of these SERS tags were not overlapped with each other. They presented the abilities for identifying and quantifying three kinds of miRNAs targets (**Figure 5**C, b). In addition, a series of studies have been conducted for multiple miRNAs detection by integrating the multiplex SERS tags with signal amplification strategies. For example, a CHA-based SERS sensor array was fabricated with CHA for target cycling and four kinds of SERS tags for targets recognition. As a result, four cancer-associated miRNAs were successfully determined in buffer, serum, and cellular extracts 39. In addition, a fluorescence-SERS dual-signal switchable probe was employed for the detection of miRNA-21 and miRNA-203 in living cells. In this assay, a 1:n ratio amplification strategy was adopted for responding to low-abundance miRNAs in living cells 109.

### Detection of proteins with SERS tags

Proteins such as prostate-specific antigen (PSA), hepatitis B surface antigen (HBsAg), and carcinoma embryonic antigen (CEA) have been used as cancer biomarkers 110. Sandwich-type immunoassays are commonly used for analyzing the species and concentrations of cancer-related protein biomarkers. The detection platforms are fabricated according to the recognition between protein biomarker and their specific antibodies. In SERS-based immunoassays, biomarkers can be recognized by immuno-functionalized SERS tags. Raman intensity and frequency are commonly used for the quantification and discrimination of biomarkers 111. By incorporating high-sensitivity SERS tags and specific immunoassays, Liu *et al*. proposed a SERS-based immunoassay for the HBsAg 112. The graphene oxide-wrapped gold nanorods as SERS tags exhibited high SERS intensity. Meanwhile, the antibodies on the SERS tags could bind HBsAg with high specificity. Both of them promoted the accurate detection of HBsAg. Serum fouling is a common phenomenon in immunoassay. To address this issue, Panikar *et al*. fabricated an anti-fouling capture SERS substrate and used it for detecting B7eH6 biomarkers in blood serum 113. In their protocol, monolayered zwitterionic L-cysteine was used to modify on a gold thin film. The fabricated substrate shows combating serum fouling properties when B7eH6 in blood serum are sandwiched by the NKp30 receptor protein on the substrate and the recognition antibody on the SERS tag, respectively.

**Figure 6:**
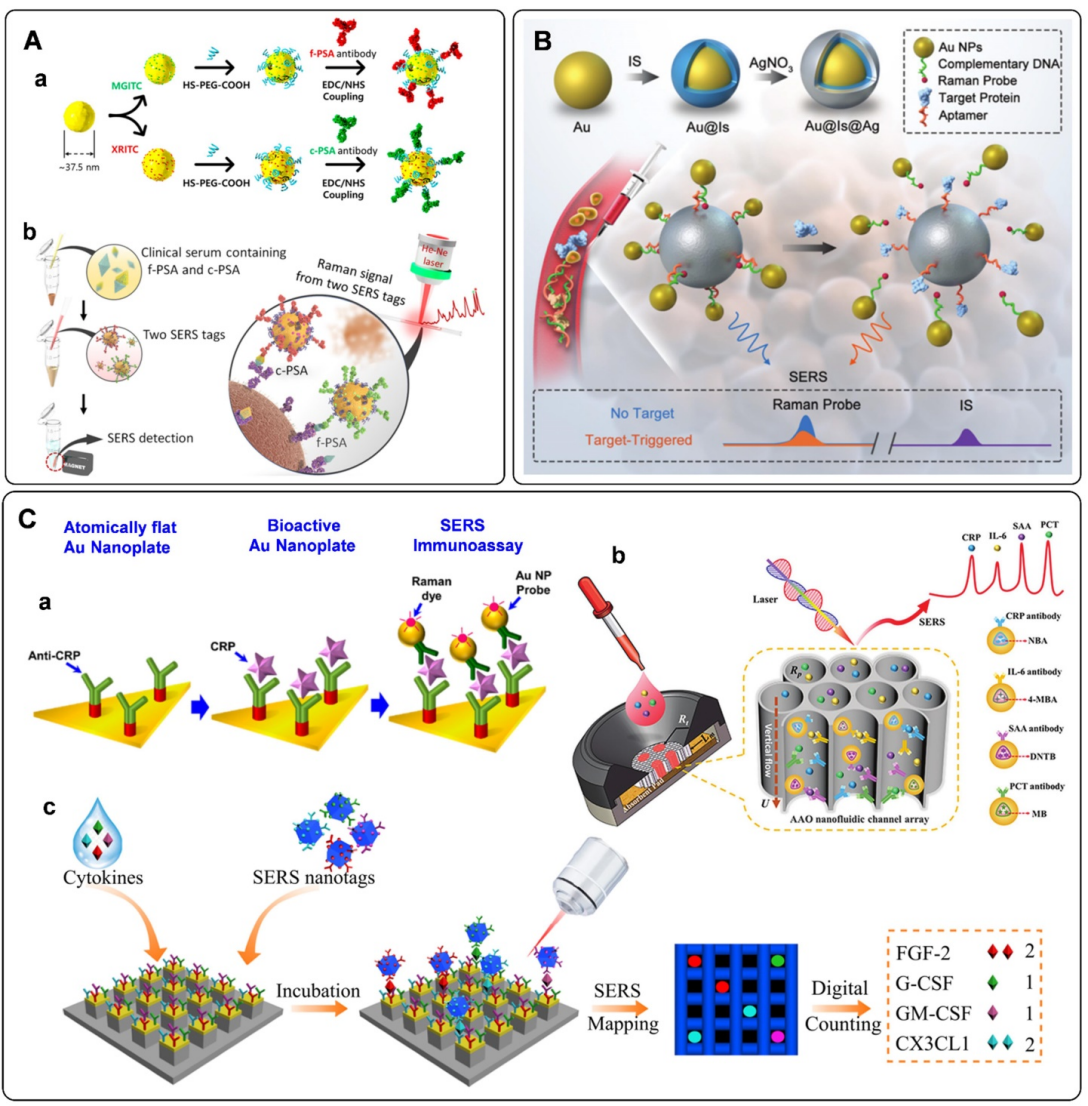
A) (a) Schematic diagram of the fabrication of SERS tags and (b) Schematic illustration of simultaneous detection of dual PSA with SERS-based immunoassay. Adapted with permission from Ref. 46, copyright 2017 American Chemical Society. B) Schematic illustration of PSA detection using an aptamer-assisted SERS sensing platform. Adapted with permission from Ref. 118, copyright 2021 Royal Society of Chemistry. C) Detection of protein biomarkers related to inflammation. (a) Schematic illustration of CRP detection using an optimally anti-CRP-immobilized Au nanoplate. (b) Schematic illustration of SERS-based multiplex vertical flow assay for the detection of four inflammatory biomarkers. (c) Digital single-molecule nanopillar SERS platform for parallel counting of four types of cytokines. Adapted with permission from Ref. 10, 47, 123, copyrights 2021 Springer Nature, 2019 American Chemical Society, 2020 Wiley-VCH.

Multiplex immuno-detection is urgent for early accurate diagnosis of cancer. The SERS platforms for multiplex protein biomarkers detection have been developed in recent years 46, 114, 115. Cheng *et al*. fabricated a SERS-based immunoassay for simultaneous detection of free PSA (f-PSA) and complexed PSA (c-PSA) 46. Antibody-modified MBs were used as the capture substrates. SERS tags functionalized with f-PSA and c-PSA antibodies were used to recognize targets in serum (**Figure 6**A). By measuring the Raman signals of the MB after immune recognition, the free to total PSA ratios can be determined to evaluate prostate cancer. Similarly, Bai *et al*. proposed a SERS immunoassay for simultaneous detection of three specific liver cancer antigens 115. Three triple bonds coded with SERS tags were used to probe three kinds of targets, respectively. When combing the enrichment effect of MB, the proposed SERS immunoassay showed the capability for rapid, sensitive, accurate, and multiplex detection of antigens.

Aside from antibodies, the cancer-related protein biomarkers can also be recognized by aptamers. Owing to their excellent binding affinity toward specific proteins, DNA aptamers have been widely employed in SERS-based immunoassays 41, 43, 116-118. With the specific recognition of the PSA aptamer and PSA target, Liu *et al*. proposed a rapid and sensitive biosensor for selective detection of PSA 117. PSA aptamers were firstly adsorbed to the surface of graphene through π - π stacking interactions. This state would be damaged when the emerged PSA bonding to its aptamer, followed by the changes of Raman frequency. The amount of PSA can be quantified by the absolute variation in frequency. Wu *et al*. designed a PSA aptamer-based biosensor from a different perspective 118. As illustrated in **Figure 6**B, an aptamer-assisted SERS sensing platform was fabricated by structure engineering with AuNPs and Au@Ag nanoparticles. The well-designed core-satellite structure with Raman probe molecules at the hot spots delivers stable Raman signals. However, they would suffer from disassembly in the presence of PSA targets. As a result, the Raman intensity would decrease. Based on this principle, a target-triggered signal-off biosensor for PSA detection has been established.

Some proteins such as C-reactive protein (CRP), serum amyloid A (SAA), procalcitonin (PCT), and interleukin-6 (IL-6) are considered to associate with the inflammation disease. Accurate and sensitive detection of these biomarkers is of great significance for the prognosis of diseases 119. A series of detection methods have been developed for inflammation biomarkers. Among them, SERS tag-based assays have been proposed and fabricated in recent years 47, 120-123. Similar to the detection of cancer protein biomarkers, sandwich immunoreactions are used to fabricate sensors for inflammatory biomarkers. As illustrated in **Figure 6**C, a, anti-CRP-immobilized plasmonic substrates were used for capturing CRP. These antigens were further recognized by CRP antibody functionalized SERS tags. According to the SERS signals derived from the Raman dyes in SERS tags, the fabricated sensor allowed specific detection of CRP at the attomolar level 47. When the above immunoassay occurs on paper substrates, SERS-based lateral flow assays are developed for inflammatory biomarkers. The protocols are the same as that of detecting DNA biomarkers with SERS-based lateral flow assays aforementioned. The characteristics of simplicity, short assay time, low cost, and flexibility for different target analytes make SERS-based lateral flow assay suitable for real-time diagnosis and point-of-care testing (POCT) of inflammation disease 121, 122. Asides from lateral flow assays, SERS-based vertical flow assays are used for detecting inflammatory biomarkers. Chen *et al*. proposed a vertical flow assay system for multiple detection of inflammatory biomarkers 123. Nanoporous anodic aluminum oxide was fabricated to support the immobilizing antibodies. Combing a 2 × 2 test array with the encoded core-shell SERS tags, four inflammatory biomarkers, including CRP, IL-6, SAA, and PCT, can be simultaneously detected with high sensitivities and wide linear dynamic ranges (**Figure 6**C, b).

Excessive inflammatory cytokines have been proved to implicate some inflammatory diseases 124. Li *et al*. developed a platform for sensitive and multiplex counting analysis of cytokines 10. The gold-topped pillar array was first prepared and functionalized with cytokine-targeted recognition antibodies. At the same time, four different dyes encoded SERS tags were applied to recognize the captured cytokines. The immunoassay of individual cytokine occurred on a single nanopillar by well-control of the target concentration. Cytokine quantification was realized by counting the number of nanopillars with specific Raman signals (**Figure 6**C, c).

Some enzymes play vital roles in life processes, and their abnormal expressions are associated with certain diseases. As a coagulation protein, thrombin is related to the diseases associated with coagulation abnormalities 125. Traditional immunoassays based on the antigen-antibody interactions are available for thrombin detection. In addition, with the discovery of thrombin aptamer, numerous aptamer-based thrombin biosensors have been developed in recent years 44, 126-128. These aptamer-based biosensors present high selectivity and sensitivity by taking aptamers and SERS tags as recognition and signals output units, respectively. Jiang *et al*. described an aptamer-based thrombin detection assay with dimeric AuNPs as SERS tags 44. The SERS tag can be captured by thrombin aptamer-modified MB through DNA hybridization. These assemblies would dissolve in the presence of thrombin. The amount of thrombin can be quantified by the Raman signals of the suspension after magnetic separation. To further improve the sensitivity of these thrombin sensors, DNA recycling amplification-assistant platforms have been developed and successfully used for detecting trace levels of thrombin 127, 128. Telomerase is considered a tumor biomarker due to its overexpression in many tumor cells. Detecting telomerase activity is of great significance for diagnosing, therapying, and monitoring cancers 129. Like thrombin detections, the SERS sensors for the determination of telomerase have been fabricated by the controllable assembly of SERS tags through DNA hybridization engineering 130-132. Besides, dual-mode platforms based on SERS and colorimetry/fluorescence were proposed to accurately determine telomerase activity in cell extracts and living cells 48, 133.

### Detection of exosomes with SERS tags

Exosomes are nanoscale extracellular vesicles originating from multivesicular bodies. They are released into the extracellular environment from most cell types, with a typical size ranging from 30 to 150 nm 2. They inherit molecular information from parental cells, such as proteins, lipids, and nucleic acids. Increasing evidence suggests that exosomes play critical roles in physiological and pathological processes as intercellular communication vectors. Consequently, they have received intense attention serving as both disease markers for diagnosis and delivery vehicles for therapy. In particular, tumor-derived exosomes have received considerable research interest due to their close relationship with cancer development, invasion and metastasis, and regulation of immune responses, making them promising as non-invasive cancer biomarkers 134. Considering the high heterogeneity, small size and low levels of exosomes in complex body fluids, there is an urgent need to develop intelligent assays for effective isolation and sensitive detection of exosomes. Until now, there are several well-established strategies, including the gold-standard method of ultracentrifugation, spin ultrafiltration, immune isolation, and liquid chromatography. However, they are usually troubled by several unsatisfactory issues, such as costly instruments, time-consuming operations, and limited accuracy.

**Figure 7:**
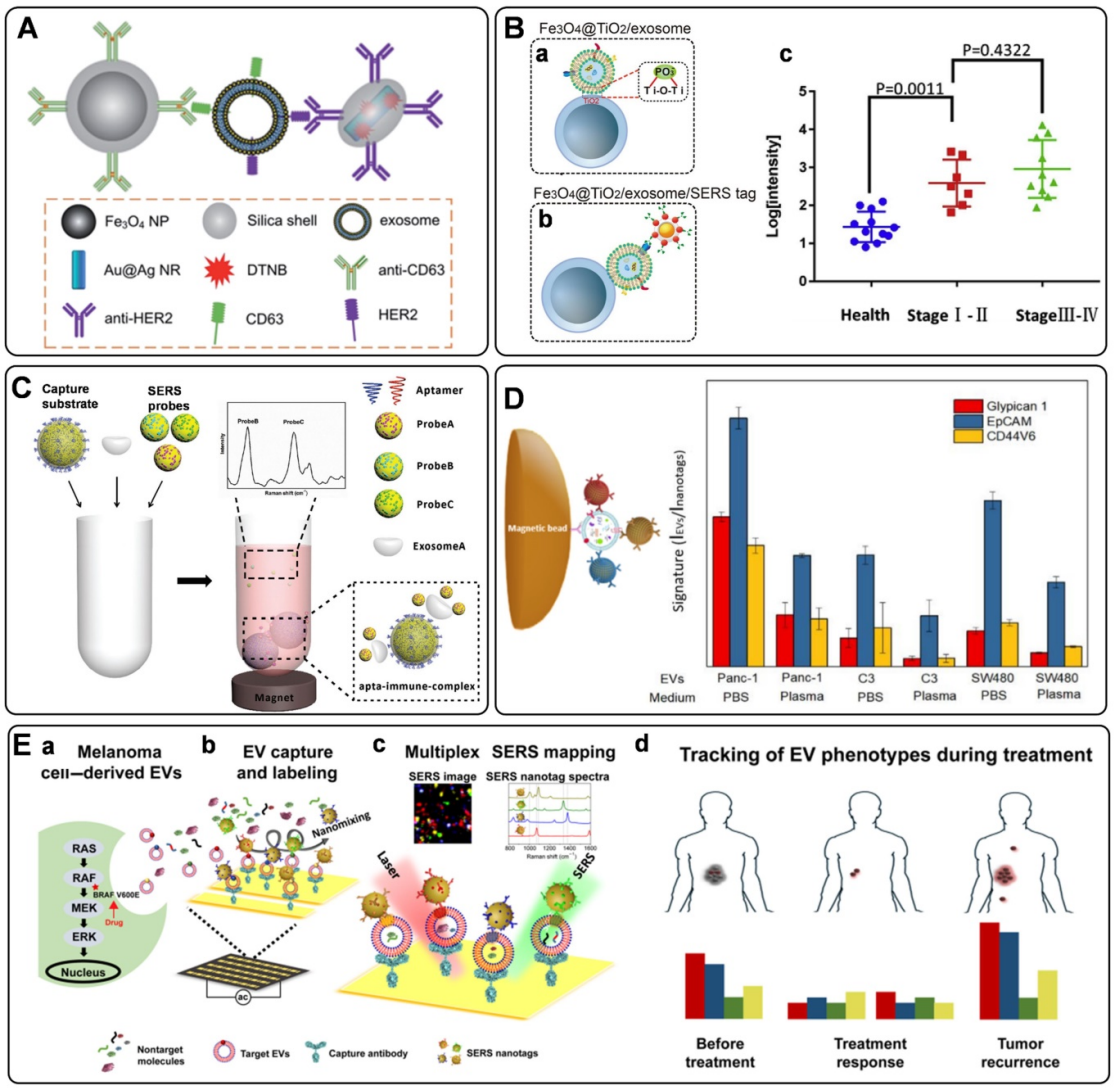
A) Sandwich-type structure formed via immune recognition between the exosome, magnetic nanobead, and SERS nanoprobe. Adapted with permission from Ref. 135, copyright 2016 Royal Society of Chemistry. B) SERS tag-based exosomal PD-L1 detection. (a) Fe_3_O_4_@TiO_2_/exosome, (b) Fe_3_O_4_@TiO_2_/exosome/SERS tag, (c) scatter plots of the log [intensity] in the serum samples from the controls and the early-stage (stage I/II) and advanced (stage III/IV) patients. Adapted with permission from Ref. 137, copyright 2020 Elsevier. C) The principle of the SERS-based detection method of multiple exosomes. Adapted with permission from Ref. 140, copyright 2018 Royal Society of Chemistry. D) Phenotypic signature of Panc-1-, C3-, and SW480-derived small EVs in PBS and plasma detected by SERS assay. Adapted with permission from Ref. 141, copyright 2020 American Chemical Society. E) Schematic for EV phenotyping by EPAC. (a) A melanoma cell with a BRAF V600E mutation secretes EVs into circulation or cell culture medium. (b) The sample is directly injected into EPAC, where a nanomixing strategy was applied to increases EV collisions with the capture antibody and SERS nanotags and shears off non-target molecules and free SERS nanotags. (c) The characterization of EV phenotypes is performed by SERS mapping. The false-color SERS spectral images are established on the basis of the characteristic peak intensities of SERS nanotags. (d) By analyzing EV samples before, during, and after BRAF inhibitor treatment, the phenotypic evolution can be tracked to provide information on treatment responses and early signs of drug resistance. Adapted with permission from Ref. 144, copyright 2021 American Association for the Advancement of Science.

Recently, optical methods for the detection of exosomes have been significantly advanced. Among them, SERS is an attractive option owing to its unique advantages of high sensitivity, specificity, multiplexing capability, and photostability 2, 134. However, SERS has only been used in the label-free analysis of exosomes until 2016, when Cui's group presented the first work to detect tumor-derived exosomes utilizing carefully designed SERS tags, illustrating the quantitative capability of labeled SERS assays for exosome detection 135. In this strategy, a typical procedure for the indirect SERS analysis of targets was followed, in which capture substrates of MBs were integrated to enable the isolation of exosomes labeled with SERS tags. Both of MBs and SERS tags are conjugated with recognition elements (commonly are antibodies) that can promote their selectivity to target exosomes. Specifically, MBs and SERS tags were modified with anti-CD63 and anti-HER2 separately to recognize two different kinds of proteins on the exosomes. With the presence of a target exosome, a sandwich-type immunocomplex can be formed between the SERS tag, exosome, and MB (**Figure 7**A). With the help of magnetics, the immunocomplexes can be isolated for SERS detection.

From then on, several similar homogeneous SERS approaches were developed for the detection of single-type exosomes 136-139. Zhang's group has used a cholesterol-modified SERS probe combined with epithelial cell adhesion molecule (EpCAM) aptamer-modified MBs for the enrichment and detection of exosomes 139. It is worth noting that a novel Raman probe with intense hot spots was prepared by assembling AuNPs in triangular pyramid DNA, providing dramatically enhanced Raman scattering. Thus, the proposed method enabled sensitive detection of MCF-7 cells-derived exosomes with a LOD of 1.1×10^2^ particles/μL. Moreover, the method could distinguish exosomes extracted from the plasma of healthy individuals and breast cancer patients. In another work, considering the low reproducibility, low exosome yield, and the biases in the exosome isolation using the immunoaffinity MBs, Pang *et al*. prepared Fe_3_O_4_@TiO_2_ NPs as capture substrates, allowing the indiscriminate isolation of exosomes through the binding of TiO_2_ and the hydrophilic phosphate head of the exosomal phospholipids (**Figure 7**B, a) 137. Then, the programmed cell death receptor ligand 1 (PD-L1) protein on the exosome was targeted with SERS tags modified with anti-PD-L1 antibodies (**Figure 7**B, b). Using exosomes derived from A549 cells as models, a detection range between 5×10^3^ to 2×10^5^ particles/mL was obtained, accompanied by a LOD of 1 PD-L1+ exosome/µL. Finally, the assay was tested with human serum samples from healthy donors and A549 nonsmall cell lung cancer patients of early and advanced stages, in which clear separation has been observed for the healthy persons and patients, whereas discrimination has not been successfully achieved for patients under different stages (**Figure 7**B, c).

Simultaneous identification of different types of exosomes could be in favor of accurate cancer detection. Considering this, Wang *et al*. have designed three kinds of SERS probes using different Raman reporters for the simultaneous detection of multiple kinds of exosomes 140. On the one hand, MBs functionalized with aptamer CD63 can capture most kinds of exosomes. On the other hand, SERS probes are modified with specific aptamers for targeting exosomes (**Figure 7**C). In the presence of the target exosomes, a sandwich structure is formed with the target exosome, MB, and the corresponding kind of SERS probes, while the other non-specific probes remain in the suspension. Consequently, a decreased SERS signal will be recorded in the supernatant, indicating the presence of the target exosomes. In addition to the multiple detection of exosomes, simultaneous profiling of multiple protein biomarkers on cancer-derived exosomes may provide much richer information of the tumor heterogeneity, facilitating the precise and accurate cancer diagnosis and cancer monitoring. In a proof-of-concept study, Zhang *et al*. realized the rapid and multiplexed phenotypic profiling of exosomes using SERS nanotags in a single test by mixing specific detection antibody-coated SERS nanotags, exosomes, and antiCD63-conjugated MBs to form a sandwich immunoassay 141. They validated the assay by detecting three surface protein biomarkers, including Glypican-1, EpCAM, and CD44 variant isoform 6 (CD44V6), to profile the molecular phenotype of Panc-1 cells-derived exosomes (**Figure 7**D). The three biomarkers were identified with three different kinds of SERS tags, composing of AuNPs modified with unique Raman reporters and conjugated with CD44V6, EpCAM, and MIL38 (specific to Glypican-1) antibodies. The sensitivity of this approach was reported as 2.3 × 10^6^ particles/mL in PBS, which is more sensitive than most other reported exosome profiling techniques. Moreover, the phenotypes of two other exosomes from a bladder cancer cell line (C3) and a colorectal cancer cell line (SW480) were also analyzed, demonstrating a significant difference in phenotypic profiles, and outlining the clear potential for further clinical applications.

Besides the homogeneous SERS assays, heterogeneous SERS assays were also fabricated for exosome detection. In the heterogeneous assays, flat supports functionalized with recognition elements for exosome binding were used instead of MBs. Very recently, a few wonderful works have been reported on the basis of the heterogeneous strategies 142-144. In 2018, Kwizera *et al*. reported a method for exosome detection and protein profiling using SERS nanotags in combination with a miniaturized capture platform 142. In this system, a gold-coated glass slide was used to develop an Au array device modified with specific antibodies to capture exosomes, and AuNRs coated with Raman reporters were used as SERS tags. Benefiting from the device, a LOD of 2×10^6^ exosomes/mL was achieved. Furthermore, in a proof-of-concept study, eight surface proteins (EpCAM, CD44, HER2, EGFR, IGFR, CD81, CD63, CD9) on model exosomes derived from breast cancer cells were analyzed. The result showed that exosomes could reflect their donor cancer cells' information, verifying the potential role of exosomes as biomarkers for cancer diagnosis. Alternatively, Wang *et al*. demonstrated an analyzer chip using a nanomixing strategy that can minimize the nonspecific adsorption and improve the capture efficiency of exosomes, especially in complex biological environments (**Figure 7**E, a-b) 144. Following this, multiplex biomarker detection is realized by simultaneously labeling the target exosomes with multicolor SERS nanotags, leading to the phenotypic evolution of exosomes (**Figure 7**E, c). Using this assay, four biomarkers are selected to monitor the exosome heterogeneity and phenotype variations of melanoma-specific exosomes, enabling the differentiation of melanoma patients and healthy individuals, as well as melanoma patients with targeted therapies (**Figure 7**E, d), which reflects the potential of exosome phenotyping for monitoring treatment responses.

### Bioimaging with SERS tags

As a unique way to “see” the micro/nano-scale biological objects within living systems, bioimaging has gained constant attention for its application in bioanalysis. Optical imaging is a charming alternative due to its capability of real-time imaging acquisition and high spatial resolution, among which fluorescence and Raman scattering are the main research focus. For a long time, bioimaging with fluorescence has been ahead of that based on Raman spectroscopy. However, along with the development of Raman instrumentations and the more and more straightforward interpretation of mechanisms and principles of SERS, SERS-active nanoprobes have illustrated optical labeling functions similar to those of fluorescence probes 8, 145. More importantly, SERS-based nanoprobes are superior to fluorescent ones for bioimaging to some extent due to their ultra-sensitivity, specificity, multiplexing, biocompatibility, and photostability. As a result, SERS tags have been used as competitive imaging agents for both *in vitro* and *in vivo* bioimaging 22. In this part, we will focus on the recent progress, mainly in the recent five years, of using SERS nanotags for bioimaging ranging from cell imaging, bacteria imaging, tissue imaging to *in vivo* bioimaging.

### Cell imaging

Cell bioimaging can supply abundant detailed information on the cellular surface biomarkers, interactions, dynamics, organelles, and microenvironments. Thus, it plays a vital role in the fundamental studies for biomedical analysis and applications. Compared to the fluorescent assays, the laser powers used in the SERS methods are much smaller, avoiding the light-induced injury of the cells and also free of photobleaching. Besides, the Raman spectra endow narrower peak widths and are more suitable for multiplex analysis. Furthermore, the laser spot of the Raman microscope is at the micrometer level (generally about 1 µm^2^), in combination with the nanoscale SERS tags, the method can provide high-resolution images of targeted cells, showing great attraction in the area for cell or subcellular imaging 146.

**Figure 8:**
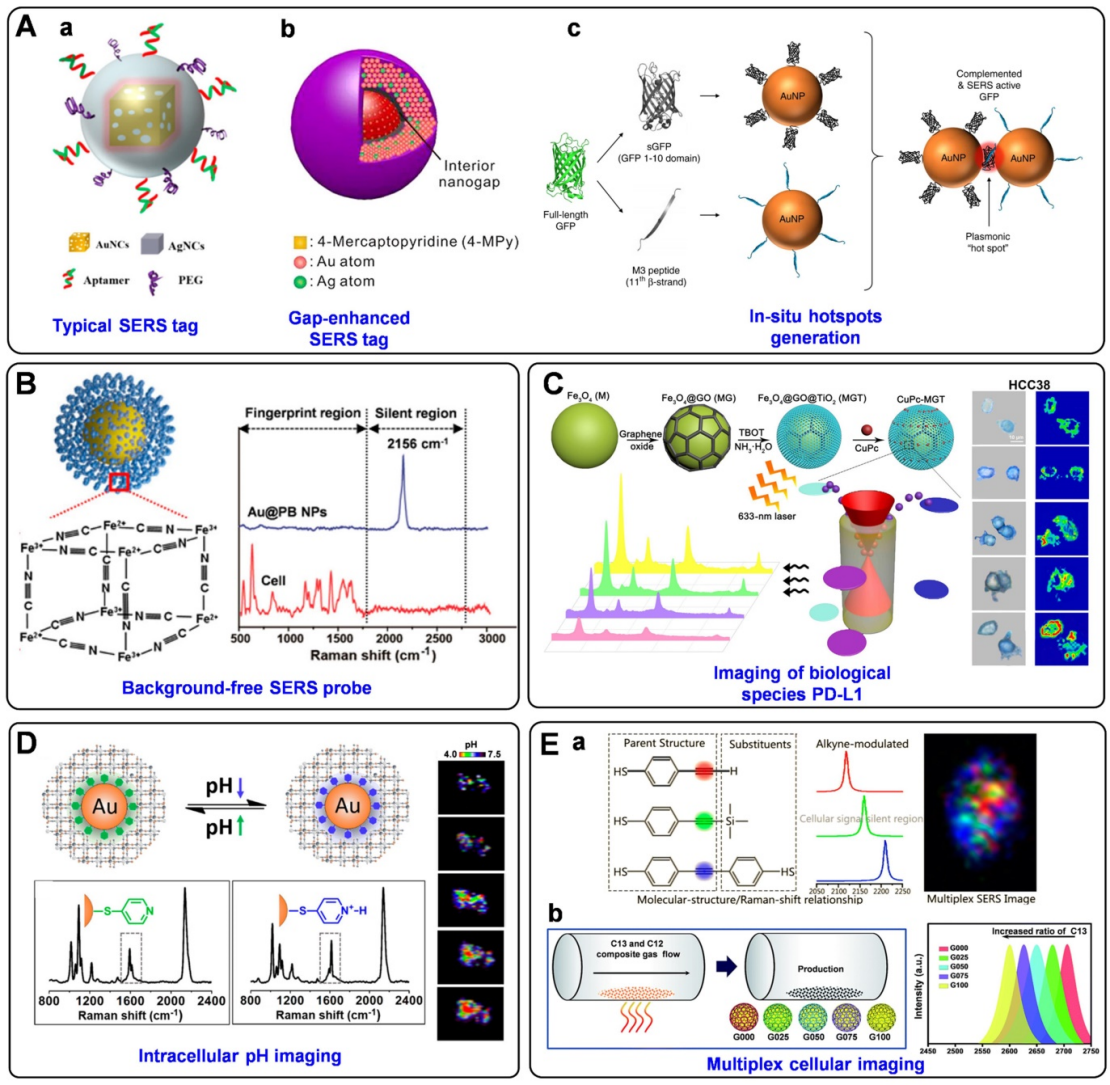
A) Different designs of SERS tags. (a) The aptamer-conjugated AuNC/SiO_2_ core-shell Raman nanoprobe. (b) The gapped DIPs. (c) Schematic of in-situ hot spots formation through self-assembly of sGFP-and M3 peptide-modified AuNPs, and GFP complementation. Adapted with permission from Ref. 147, 148, 150. copyrights 2019 American Chemical Society, 2018 American Chemical Society, 2018 Springer Nature. B) The structure of Au@PB NPs and the Raman spectra of Au@PB NPs and HepG2 cells. Adapted with permission from Ref. 74, copyright 2017 American Chemical Society. C) Schematic illustration of the synthesis process and enhancement mechanism of the MGT substrate. Right panel: Raman mapping images toward PD-L1 obtained from HCC38 cells incubated with IFN-γ. Adapted with permission from Ref. 156, copyright 2018 American Association for the Advancement of Science. D) The sensing mechanism of the PB-caged SERS probe, and the real-time pH imaging of a single cell. Adapted with permission from Ref. 160, copyright 2020 American Chemical Society. E) (a) The molecular structures of 4-ethynylbenzenethiol derivatives and the corresponding Raman shift and a three-color SERS image of a single live Hela cell. (b) Synthesis strategy as well as the SERS spectra of multiplexed SERS tags by tuning the ratiometric composition of C13 and C12. Adapted with permission from Ref. 163, 164, copyrights 2016 American Chemical Society, 2018 Royal Society of Chemistry.

One dominating use of SERS tags in cells is accurate imaging of the cellular biomarkers, which is highly critical for high-throughput screening of targeted cells for better understanding the related biological events. Wen *et al*. designed a bifunctional probe using Au nanocage as SERS-active substrate, 4-MBA as Raman reporter, SiO_2_ as a protective shell, and aptamer AS1411 as recognition unit (short as AuNC/SiO_2_/Apt) (**Figure 8**A, a) 147. This proposed nanotag endows high stability and biocompatibility due to the SiO_2_ coating, and has been applied as a novel theranostic platform for targeted SERS imaging and PTT of nucleolin-overexpressing cancer cells. To further enhance the brightness of SERS probes, SERS-active substrates with rich hot spots have been fabricated. For example, ultrabright gap-enhanced SERS tags have been developed by Nam's 148 and Ye's groups 149 for cell imaging. In Nam's study, dealloyed intra-nanogap particles (DIPs) with a ∼2 nm intragap filled with Raman reporters (**Figure 8**A, b) have been prepared, displaying a very narrow distributed and high enhancement factor (≥1.1×10^8^). By functionalizing the DIPs with cell-targeting cRGD, *in situ* long-term real-time SERS imaging has been achieved of integrin α_ν_β_3_ expression cells in a highly reliable and quantifiable manner. Furthermore, cellular imaging by the target-triggered assembly of hot-spot SERS nanoprobes in living cells has also been demonstrated 53, 150. Koker *et al.* used split-fluorescent protein fragments as molecular glue and switchable Raman reporters to assemble plasmonic NPs into photonic clusters (**Figure 8**A, c) with homogeneously distributed hot spots directly in live cells 150. The autocatalytic activation of the fluorescent protein chromophore and near-field amplification of its Raman fingerprints within the hot spots enable the selective and sensitive SERS imaging of targeted cells.

In addition to improving the brightness, background-free SERS tags have also been fabricated for bioimaging. Such SERS tags possess characteristic peaks in the Raman-silent region, where no Raman signals can be recorded from the endogenous biomolecules 151. In the past five years, our group has designed a series of smart SERS probes in the Raman-silent region with high SBR for bioimaging 74, 152-155. In 2017, we presented a surprising discovery that PB can be assembled onto AuNPs (named as Au@PB) to serve as SERRS tags **(Figure 8**B). On the one hand, PB only exhibits a strong and sharp single peak at 2156 cm^-1^ (low background). On the other hand, the UV-vis absorption of PB is in resonance with the incident laser (high signal). As a result, PB has been employed as a Raman reporter with high SBR for the first time, which not only promoted the sensitivity of the tags but also endowed imaging with high specificity in biological samples 74. Later, the brightness of PB-based Raman probes has been further enhanced by assembling PB onto porous AuNPs bearing intense hot spots, which has been used to profile folic acids (FA) at a single-cell level 152. Additionally, a core-shell nanostructure with embedded Raman reporters 4-MBN has been prepared for single-cell molecular imaging 153. And an alkyne-bridged plasmonic dimer SERS probe was fabricated for high precision profiling of sialic acid (SA) expression both in cancer cells and tissues 154. Moreover, pyrophosphate imaging in living cells has been achieved due to the *in-situ* NPs dimerization triggered by intracellular pyrophosphate, which generated intense electromagnetic hot spots and dramatically enhanced the SERS signals of Raman reporter 4-MBN 155. All these probes exhibit strong and sharp single peaks in the cellular Raman-silent region, thus eliminating the possible background interference and displaying high-precision bioimaging capability at the single-cell level.

SERS tags are also powerful tools for determining trace cellular biological species as well as changes in the cellular microenvironment. For instance, PD-L1 156, cholesterol 157, reactive oxygen species (ROS) 158, and caspase-3 159 in cells have all been successfully imaged based on the labeled SERS technique. Feng *et al*. synthesized innovative SERS probes using a ternary heterostructure of Fe_3_O_4_@GO@TiO_2_ (denoted as MGT) as plasmon-free SERS substrate and CuPc as Raman reporter 156. Thanks to the resonance effect of CuPc and the efficient charge transfer between CuPc and MGT, the as-prepared probe presented the remarkable enhanced effect of SERS signal, which has been successfully applied for *in situ* quantification and imaging of PD-L1 at the single-cell level and for monitoring the dynamic change of PD-L1 during drug treatment (**Figure 8**C). Very recently, Zhu *et al*. prepared two different triple bond-labeled AuNPs, which can be triggered by caspase-3 to serve as “click” SERS probes with hot spots. To this end, precise intracellular imaging of caspase-3 can be *in situ* monitored in living cells or during cell apoptosis 159.

What's more, the cellular parameters that can reflect the intracellular microenvironment, such as pH 67, 160, 161 and hypoxia 162, have also been measured and imaged by monitoring and analyzing the signal variations of the SERS tags. Intracellular pH is an important modulator that is highly associated with cell functions, and the reliable quantification and imaging of pH variations in live cells are of great importance for understanding the related physiological processes. Our group reported a robust PB-caged pH-responsive SERS probe for precisely imaging the dynamic pH changes in live cells 160. Utilizing PB as a bi-functional unit, which can not only protect the probes from interfering substances but also serve as a background-free IS, the as-designed probes can reliably determine the dynamic pH changes associated with autophagy under diverse conditions (**Figure 8**D). Moreover, a ratiometric SERS nanoprobe for imaging hypoxic living cells or tissues has been proposed by assembling azo-alkynes on an Ag/Au-modified single-walled carbon nanotube 162. In this probe, the intensity of the alkyne Raman band (2207 cm^-1^) is target-dependent, and the 2D-band of SWCNTs (2578 cm^-1^) is used as IS, both of which lie in the cellular Raman-silent region. By combining with the anti-interference property, this novel ratiometric SERS assay has shown promising application for both *in vitro* and *in vivo* imaging of hypoxia.

Simultaneous detection and imaging of multiple cancer-related biomarkers on the cell surface could provide invaluable information for accurate cancer diagnostics and improve the clinical potential. To perform such multiplexed cellular imaging, Shen and Hu *et al.* constructed an alkyne-modulated SERS palette by rationally designed 4-ethynylbenzenethiol derivatives 163, which exhibited narrow emissions in 2100-2300 cm^-1^, avoiding the optical interference originating from the lower wavenumber region (<1800 cm^-1^). Using Au@Ag NPs as enhancement substrates and the derivatives as Raman reporters, three kinds of targeting SERS tags were prepared for interference-free multiplex SERS imaging of live cells (**Figure 8**E, a). Later, they also outlined a novel readout technique, so-called “Click” SERS, based on triple bond-containing reporters-encoded SERS-active NPs 54. Different from the conventional “sole code related to sole target” readout protocol, the “Click” SERS relies on the number rather than the intensity of combinatorial emissions. With this technique, accurate cellular imaging under double exposure has been achieved. Furthermore, Zou *et al.* reported a ratiometric control of C12 and C13 isotopes to fabricate multicolor isotopic graphene-isolated-Au-nanocrystals, by shifting the Raman 2D-band of graphene from 2600 to 2706 cm^-1^ (**Figure 8**E, b) 164. Such tags endowed non-overlapped characteristic Raman bands in the cellular Raman-silent region, demonstrating multiplexed Raman imaging and pattern recognition of targeted cancer cells after the conjugation with cell-specific aptamers.

### Bacteria imaging

Bacteria detection and screening have received increasing attention due to the huge demand in public health, clinical diagnosis, and the food industry. SERS-based bacterial assays can be divided into two categories: the direct way and the indirect way. The former uses the intrinsic SERS signals of bacteria, and the latter needs the help of identification unit-conjugated SERS nanotags 165. In a long-lasting time until now, most of the Raman-based bacterial assays have been focused on analytical applications. There are very limited research activities in studying the uses of Raman methods for bacteria imaging, especially the indirect assays. It should be pointed out that, unlike the mammalian cells, bacterial cells have a much smaller size comparable to that of the Raman laser spot. Thus, generally, bacterial cells can only be imaged by the SERS technique as a whole, barely supplying more specific and detailed molecular information. This may be the main reason why the application of SERS in the area of bacteria imaging has fallen behind that of mammalian cell imaging.

**Figure 9:**
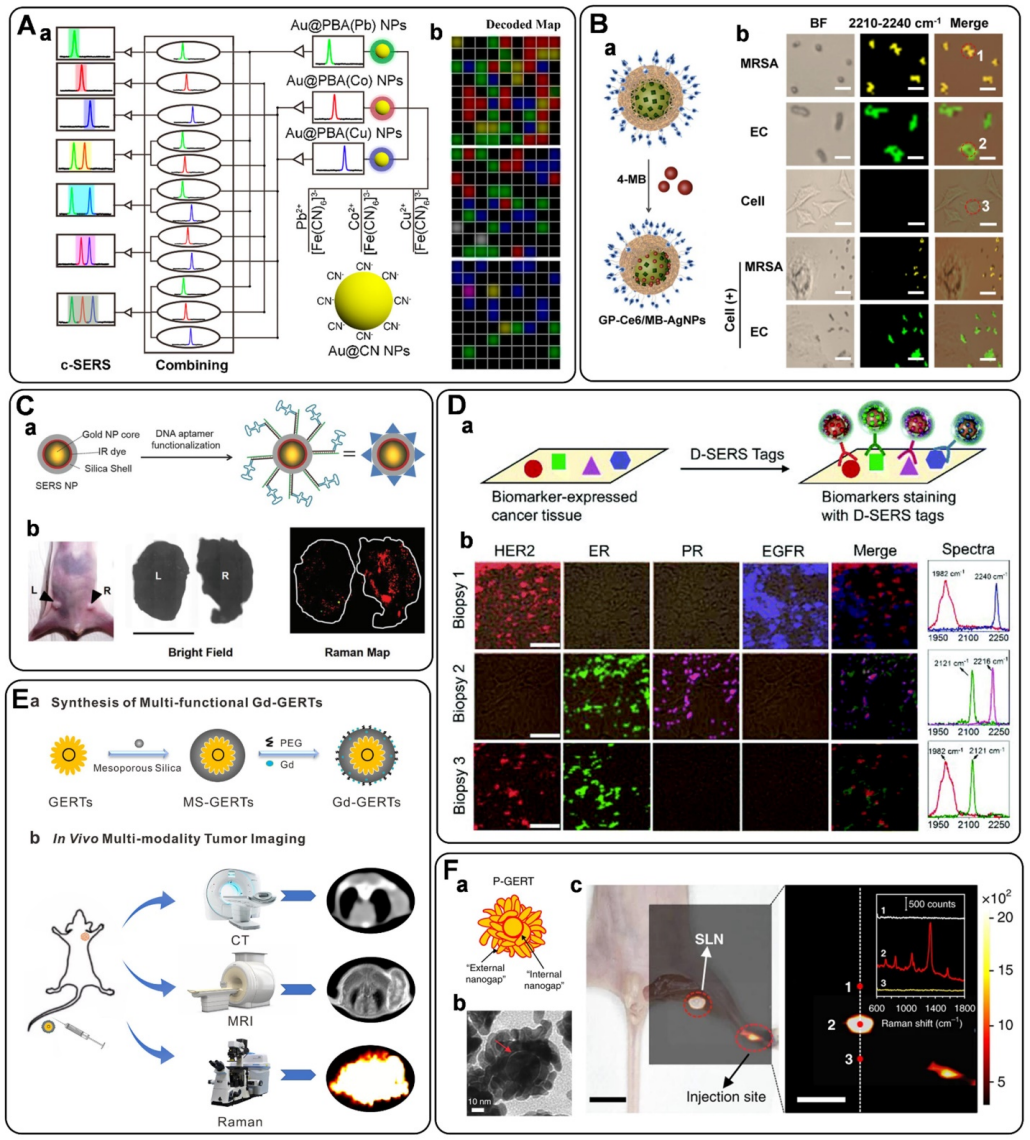
A) (a) Schematic demonstration of the conception of the combined SERS emissions (c-SERS). (b) Representative decoding image of four common bacteria by means of c-SERS. Adapted with permission from Ref. 168, copyright 2019 American Chemical Society. B) (a) Schematic illustration of GP-Ce6/MB-AgNPs. 4-MBN was introduced on the surface of AgNPs during the PDA polymerization process. (b) Raman mapping images in the nitrile channel (2228 cm^-1^) for GP-Ce6/MB-AgNPs on MRSA, EC, 3T3 cell, MRSA + 3T3 cell, and EC + 3T3 cell. Adapted with permission from Ref. 171, copyright 2021 Ivyspring. C) (a) Fabrication of SERS NPs, consisting of AuNP core, IR dye coating, and silica shell, and MUC1 DNA aptamers. (b) Left: photograph of a nude athymic mouse with MDA-MB-468 tumor (R) and MDA-MB-453 (L) xenograft. Middle: Bright field image of the excised tumors. Scale bar 5 mm. Right: *Ex vivo* Raman image of the tumors. Adapted with permission from Ref. 57, copyright 2017 Wiley-VCH. D) Multiplexed Raman imaging of tumor biomarkers in breast cancer biopsies from three patients. (a) Schematic of the multiplexed biomarker imaging. (b) Raman mapping images of four pseudo-color (red, green, magenta, and blue) channels correspond to SERS tags targeting HER2, ER, PR, and EGFR, respectively. Scale bar = 50 µm. Adapted with permission from Ref. 65, copyright 2018 Royal Society of Chemistry. E) (a) The synthesis process of Gd-loaded GERTs (Gd-GERTs). (b) their application for *in vivo* CT/MRI/Raman multimodality tumor imaging. Adapted with permission from Ref. 182, copyright 2020 Elsevier. F) Schematic diagrams (a) and representative TEM image (b) of p-GERTs. (c) High-contrast and wide-area *in vivo* Raman image (3.2 × 2.8 cm^2^) of the hind-limb popliteal lymph node after injection of P-GERTs. Adapted with permission from Ref. 184, copyright 2019 Springer Nature.

A typical procedure for the SERS imaging of bacteria usually involves three steps: bacteria capture, labeling (resulting in a typical sandwich configuration), and Raman mapping. Ko *et al.* prepared a poly (L-lysine)-coated plasmonic substrate for bacteria capture. After selective labeling of the bacterial surfaces with antibody-conjugated SERS nanotags, Raman mapping was conducted to image the labelled bacteria. More importantly, the mapping data have been further analyzed for quantitative analysis of bacteria, realizing the reliable SERS imaging-based detection of bacterial pathogens 166. This method has been further expanded by Liu's group 167, in which Au@PB NPs possessing a characteristic peak in the Raman-silent region have been sent as SERS tags to indicate the presence of target bacteria. Benefiting from the anti-interference capability of Au@PB, a model bacterium of *Staphylococcus aureus* has been successfully detected and imaged in the whole blood samples. Speaking of Au@PB, Hu's group has presented a creative idea to tune the SERS emissions of Au@PB by simply replacing the original Fe^2+^/Fe^3+^ with other metal ions (i.e., Pb^2+^, Co^2+^, Cu^2+^) (**Figure 9**A, a). By incorporating with bacteria-specific aptamers, the as-prepared three distinct SERS tags have advanced a super-multiplex bacteria barcoding model to an encoding capacity of 2^n^ -1, demonstrating the simultaneously multiplex imaging of micrometer-sized objects (**Figure 9**A, b) 168.

Back to the above-mentioned work reported by Liu's group 167, the Au@PB NPs have been designed not only as a SERS tag but also as an antibacterial agent due to their photothermal effect. Earlier, Jiang's group fabricated a 2,3-naphthalocyanine dihydroxide- and vancomycin-modified silica-encapsulated, silver-coated AuNPs for SERS imaging and antimicrobial PDT (aPDT) of vancomycin-resistant *enterococci* strains 169. In another example, they proposed an approach based on bacteria-induced AuNPs aggregation, in which rich hot spots and strong NIR effects were generated, enabling the effective SERS imaging and photokilling of bacterial pathogens 170. All these works show that it is a fashion manner to develop smart SERS tags with multifunction for bacteria sensing, imaging, and elimination. Lately, our group has illustrated an integrated theranostic system based on photoactive silver nanoagents for monitoring, imaging, and precision killing of drug-resistant bacteria 171. Nowadays, drug-resistant bacteria account for numerous treatment failures and mortality in the clinic 165. Thus, it is an urgent need to develop effective strategies to identify and eliminate them. In this assay, AgNP was decorated with photosensitizers (Chlorin e6, Ce6), 4-MBN (Raman reporter), and bacteria-specific units of Poly[4-O-(α-D-glucopyranosyl)-D-glucopyranose] (GP) as a multifunctional probe (short as GP-Ce6/MB-AgNPs) (**Figure 9**B, a). Using Methicillin-resistant *Staphylococcus aureus* (MRSA) and *Escherichia coli* (EC) as a model bacterium, upon 655 nm laser activation, Ce6 can produce ROS to induce the rapid release of Ag^+^ from the AgNP core to kill bacteria. Additionally, the AgNP modified with 4-MBN can image the labeled bacteria (**Figure 9**B, b) and monitor their metabolic dynamics in the infection sites, showing great potential for detection, imaging, and elimination of drug-resistant bacteria in diverse clinical settings.

Furthermore, 3,3'-diethylthiatricarbocyanine iodide (DTTC)-conjugated gold-silver nanoshells (AuAgNSs-DTTC) were prepared by He *et al.* for detection and imaging of drug-resistant bacteria both *in vitro* and *in vivo* 172. On the one hand, under NIR laser, the efficient photothermal effect and the simultaneously released Ag^+^ from AuAgNSs-DTTC could eradicate bacteria. On the other hand, AuAgNSs-DTTC enabled the in-situ SERS imaging of bacteria on the infected wound area to provide a non-invasive and prolonged tracking platform for imaging the residual bacteria. This multifunctional AuAgNSs-based nanosystem is a promising diagnosis and treatment tool for clinical use against bacterial infections.

### Tissue imaging

SERS tags have proven to be excellent labels for tissue imaging. The crucial advantages include the low interference from biological matrices, high photostability, high spatial resolution, and multiplexing capability. In 2006, SchlÜcker *et al.* demonstrated the first realization of SERS tags for *in situ* antigen imaging in tissue samples 173. By incubating the PSA antibody-modified SERS tags with the tissue samples, the localization of PSA in epithelial tissue was monitored based on the characteristic Raman signals of SERS labels. Recently, a similar strategy has been utilized for single-color SERS imaging in cancer tissues 57, 154, lymph node tissue 174, and liver tissue 162, by getting helping hands from carefully designed novel SERS tags. In detail, Pal *et al.* reported the development of a DNA aptamer modified SERS NPs (**Figure 9**C, a) for imaging of Mucin 1 (MUC1) overexpression in human breast cancer 57. After injecting SERS probes via tail vein for 16-18 h into tumor xenograft mouse models, the tumors were excised, and the MUC 1 imaging was carried out *ex vivo*. Clear evidence suggested that the SERS probes homed to the tumors via active targeting of MUC1, which was expected as an advantageous alternative for targeted imaging of tumor extent, progression, and therapeutic response (**Figure 9**C, b). In another example, our group has synthesized alkyne-bridged AuNP dimers conjugated with PBA to profile the SA expression in cells and tissues 154. The as-constructed SERS probes of AuNP dimers exhibited a high SBR with enhanced background-free SERS signals in the Raman-silent region, realizing the accurate profiling of SA expression on cancer tissues with different metastasis degrees. Subsequently, we fabricated gadolinium-doped Au@PB NPs as magnetic resonance imaging (MRI)/SERS bimodal probes for dendritic cell activating and tracking 174. After the modification with ovalbumin, the as-prepared probe was used for real-time tracking of the dendritic cell migration process by MRI. Moreover, the distribution of the colonized dendritic cells in the lymph node tissue slice was profiled using the Raman mapping technique based on the characteristic background-free band of PB.

In addition to the typical single-color imaging of tissue samples, as mentioned above, a SERS probe named SWCNT/Ag/AuNPs has been presented for ratiometric SERS imaging of hypoxia both in living cells and in rat liver tissue samples derived from hepatic ischemia surgery 162. Song and co-workers developed a novel core-satellite gold nanostructure for dual ratiometric SERS and photoacoustic (PA) imaging of inflammation/cancer-related H_2_O_2_ 175. The oxidative species produced in inflammation, tumor, and osteoarthritis in rabbits can cause the dissociation of the gold nanostructure, resulting in ratiometric changes of the SERS and PA signals of the probe, which enabled the differentiation between the inflamed region and normal tissue with high accuracy. Except for the target-dependent SERS imaging, SERS tags have also been used as a nanomedicine to evaluate the enhanced permeation and retention (EPR) of NPs in spontaneous brain tumors 176. Considering the similarities between brain tumors in humans and dogs, canine brain tumors were used as models to investigate the distribution of PEGylated silica-coated, Raman reporter-embedded gold nanoparticles in different brain tumor pathologies using SERS. SERS imaging of the resected tumor tissues proved the heterogeneous EPR of nanoparticles into oligodendrogliomas and meningiomas of different grades, while no detectable traces were recorded in necrotic parts of the tumors or normal brain. Such heterogeneities should also be considered to accelerate the clinical management of brain tumors using nanomedicine approaches.

Molecular imaging of multiple overexpressed tumor-related biomarkers on a single tissue sample is an approach with the potential to provide more accurate information for tumor diagnosis. In this regard, multiplex SERS imaging of different kinds of biomarkers simultaneously in tissue samples, including two-color 177, 178, three-color 40 and four-color 65, 179, 180 bioimaging assays, have also been demonstrated using multicolor SERS tags. Earlier, SchlÜcker's group realized the two-color imaging of p63 and PSA on prostate tissue based on two different SERS labels comprising silica-coated gold nanoparticle clusters and Raman reporters 177. Lately, Eppe and co-workers stated a similar strategy to image folate receptors and SA simultaneously in breast and cancerous ovarian tissues, in which two types of SERS tags using Au@Ag as plasmonic substrate, and malachite green isothiocyanate (MG ITC) and oxazine170 as Raman reporters were applied 178. We have presented an assay for multiplex SERS imaging of tissue samples by using alkyne- and nitrile-bearing molecules to directly fabricate three types of SERS tags, which all appeared strong and nonoverlapping signals in the cellular Raman-silent region 40. After the modification with different antibodies toward different biomarkers (ER, EGFR, PR), multicolor imaging of cancer cells and human breast cancer tissues has been completed, confirming their great potential for multiplex imaging in clinical diagnosis. In another work, four non-overlapping SERS tags have also been synthesized in our group via a universal one-pot synthesis strategy, followed by the screening of total 82 background-free Raman reporters 65. For a proof-of-concept study, multicolor imaging of four cancer biomarkers (HER2, ER, PR, and EGFR) in breast cancer biopsies from three patients has been successfully demonstrated (**Figure 9**D). It is also worth mentioning that Liu and co-workers have performed the multiplexed molecular imaging of freshly excised breast tissues with a mixture of five flavors of SERS NPs (four targeted and one untargeted control), which have been functionalized with corresponding antibodies to simultaneously target EGFR, HER2, CD24 and CD44 179, 180.

### *In vivo* imaging

Beyond imaging of *in vitro* cellular, bacterial, and tissue samples, SERS imaging with SERS tags has been further extended to the* in vivo* analysis. Optical imaging of living subjects is highly required in many fields, such as biomedical research and patient treatment. Among the various imaging modalities, including SERS, fluorescence, chemiluminescence, and PA, SERS has emerged as a novel and powerful tool for optical imaging* in vivo* 22. For instance, compared to the fluorescent assays, multiplexing is easily performed with multicolor SERS tags. The anti-photobleaching capability of SERS tags makes them suitable for* in vivo* studies of prolonged duration. Moreover, the biosafety of SERS tags is more reassuring due to the commonly used plasmonic substrates of Au. Therefore, *in vivo* SERS imaging has a bright future in real-world applications.

SERS tags were used for *in vivo* tumor targeting for the first time in 2008 181. Nie and co-workers have demonstrated a SERS tag composed of 60 nm Au, Raman reporter of crystal violet, and a layer of thiol-PEG. When conjugated to tumor-targeting ligands of single-chain variable fragment antibodies, the SERS tags have realized the *in vivo* tumor detection in a xenograft tumor model. Following this pioneered work, much progress has been made in recent years, especially the series of works presented by Ye and co-workers 61, 182-184. They implemented the *in vivo* bioimaging of tumors 61, 182 and lymph nodes 183, 184 with rational designed gap-enhanced Raman tags (GERTs). For example, mesoporous silica- (MS-) coated GERTs (MS GERTs) were designed by coating the Au core with a layer of Raman reporters of 1,4-benzenedithiol (BDT), which can further guide the growth of the Au shell and the external MS layer 61, 183. The as-prepared Raman tag exhibited ultrahigh photostability because of the off-resonance between the built-in Raman reporters and laser excitation, and ultra-brightness due to the combination of the chemical and electromagnetic enhancement effects originating from the sub-nanometer core-shell junction. As a result, these MS GERTs achieved continuous and stable imaging of orthotopic prostate tumors in mice models 61. In a subsequent study, the same probe was used to visualize sentinel lymph nodes (SLNs) in living mice. The obtained Raman image exhibited strong Raman signals of GERTs at the SLN position, while no signals were recorded from the surrounding tissues, suggesting a great potential for clinical use 183. Later, Ye and co-workers have synthesized similar GERTs by replacing the BDT with a new Raman reporter of 4-nitrobenzenethiol (4-NBT) and doping gadolinium into the MS layer. The probe exhibited excellent capabilities for computed X-ray tomography (CT), MRI, and SERS imaging (**Figure 9**E). In particular, the *in vivo* intraoperative SERS imaging of tumors can delineate the tumor margin, and thus, can guide the surgery of tumor excision 182.

The existing SERS imaging speed lags far behind practical needs, mainly limited by Raman signals of SERS nanoprobes. To this end, a version of ultrabright GRETs with petal-like shell structure (P-GERTs) was presented by Ye's group 184. The as-proposed P-GERTs possessed a high enhancement factor beyond 5×10^9^, benefiting from the strong electromagnetic hot spots from the gaps and the petal-like shell, the larger surface area for molecular immobilization, and a larger Raman cross-section of reporter molecules (4-NBT) (**Figure 9**F, a-b). Finally, the P-GERTs have been used to realize the high-contrast and wide-area *in vivo* Raman imaging of the hind-limb popliteal lymph node within 52 s (**Figure 9**F, c). To sum up, these GERTs offer a potential solution to overcome the current bottleneck in the field of SERS-based bioimaging. It is worth noting that *in vivo* bioimaging based on SERS tags has also played an essential role in biomedical intraoperative applications, especially for cancers, which will be discussed in detail in the following part.

### SERS tag-guided tumor therapy

In the past decade, SERS has progressed from proof-of-concept studies to point-of-care applications in clinical oncology. It now appears that the most important factor that contributes to this transformation of the SERS technique in the medical field has been the introduction of SERS tags. In addition to the application in cancer diagnosis, SERS tags have also displayed increasingly critical roles in cancer therapy. On the one hand, accumulating evidence has suggested that SERS tags have emerged as a new image-guidance tool to help surgeons precisely and sensitively identify tumor margins for surgical resection. On the other hand, SERS-guided theranostic platforms have received considerable attention based on smart multifunctional SERS tags. All this progress reveals that SERS tags could serve as the next-generation promising tool for personalized cancer diagnosis and treatment 6.

### Delineating the tumor margin

Once cancer is diagnosed, surgery remains the mainstay for treatment, especially for most solid tumors. However, surgeons face challenges in intraoperatively identifying tumor margins due to their infiltrative nature, whereas incomplete surgical resection may lead to early recurrence, and excessive resection may injure adjacent functional tissues. Moreover, cancer may be multifocal and characterized by the presence of microscopic satellite lesions. Such microscopic foci are one of the major reasons for local recurrences and cancer metastasis and are usually hard to be visualized via current imaging techniques. Therefore, intraoperative imaging guidance that enables more precise delineation of tumor margins, as well as detection of invisible microscopic lesions, is urgently needed in the surgery-based strategies for cancer treatment. Recently, this goal has been facilitated via the use of SERS tags. This conclusion has been supported by the excellent works done by several research groups, including Kircher and co-workers 15, 18, 185-189, Liu group 190, 191, and Li and co-workers 16, 17, 192.

**Figure 10:**
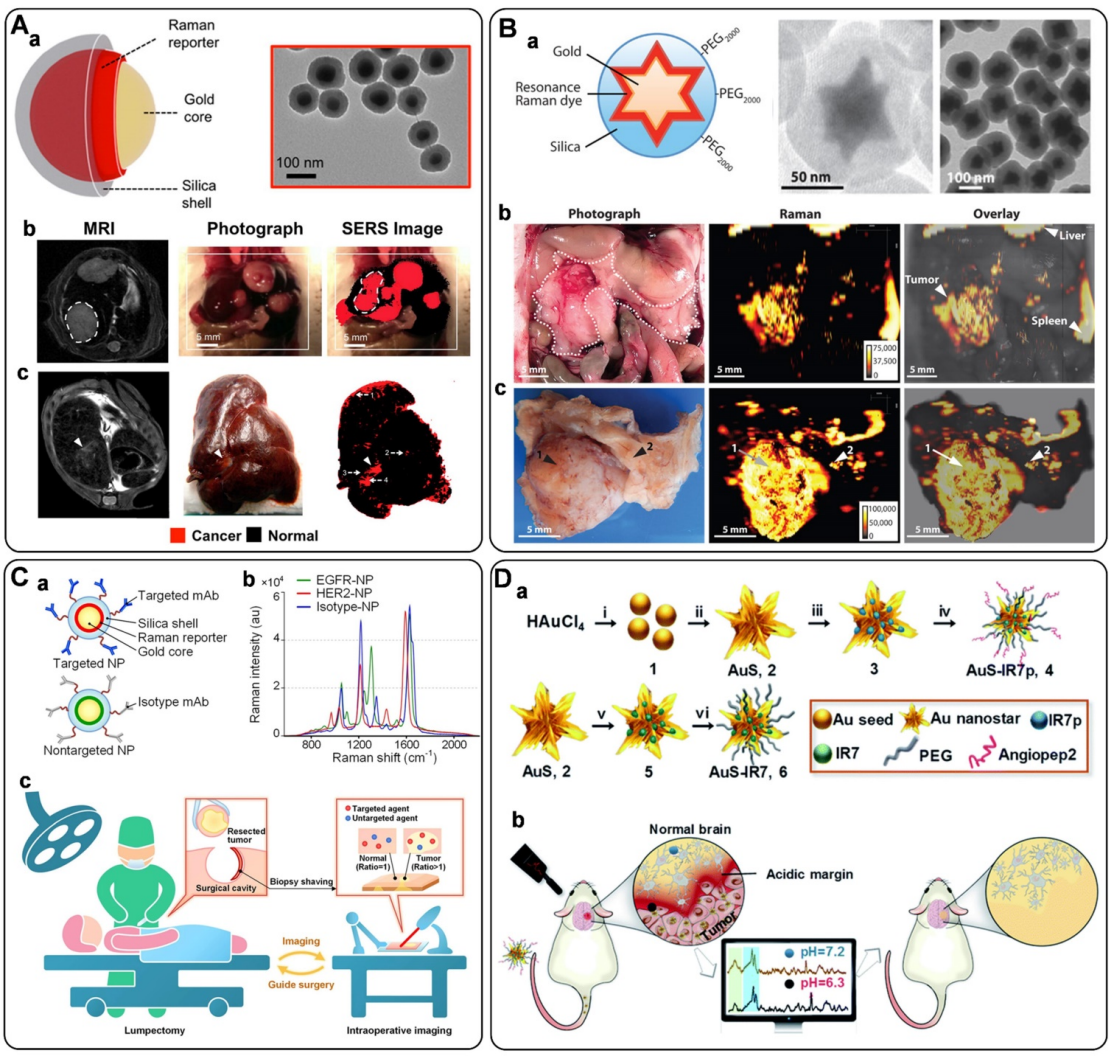
A) (a) Illustration and the corresponding TEM image of the SERS NPs. MRI, photograph, and *in vivo* SERS image of liver tumors (b, genetic Myc-driven HCC mouse model) and microscopic liver tumors (c, histiosarcomas; genetic Ink4A/Arf^-/-^ mouse model). Adapted with permission from Ref. 15, copyright 2016 American Chemical Society. B) (a) Illustration and the corresponding TEM image of the SERRS nanostar. (b) *In situ* photograph of the exposed upper abdomen in a mouse with a pancreatic ductal adenocarcinoma in the head of the pancreas (outlined with white dotted line). Corresponding Raman images, showing SERRS nanostar signal in the macroscopically visible tumor in the head as well as small scattered foci of SERRS signal in other normal-appearing regions of the pancreas, are also shown. (c) Photographic and high-resolution Raman images of the excised pancreas from (b). Adapted with permission from Ref. 187, copyright 2015 American Association for the Advancement of Science. C) (a) A depiction of the structure of the targeted and nontargeted SERS NPs and (b) the Raman spectra of the SERS NPs (targeted and nontargeted) used in this study. (c) Schematic of an intraoperative imaging technique to rapidly identify residual tumors at the margins of freshly resected tissues for guiding breast-conserving surgeries. Adapted with permission from Ref. 191, copyright 2016 Springer Nature. D) (a) Synthesis of pH-responsive SERRS probe AuS-IR7p and the control probe AuS-IR7. (b) Illustration presenting the acidic margin-guided brain tumor surgery by intra-operatively determining tissue pH values/malignancies in tumor cutting edges. Adapted with permission from Ref. 17, copyright 2020 Royal Society of Chemistry.

In 2012, Kircher and co-workers synthesized their first generation of SERS tags by coating a Raman active layer and a silica shell onto the surface of a gold core. By using a static SERS imaging microscope, the SERS probe has been served as an image-guidance tool to help in identifying microscopic tumors in implanted glioma mouse models 185. Later, considering the fact that real-time SERS imaging in the operating room is difficult to realize due to the lack of rapid wide-area SERS imaging devices, Kircher and co-workers fabricated a new strategy by combining static SERS imaging with near real-time SERS detection based on a hand-held Raman scanner, in an intraoperative scenario 186. Microscopic foci at the resection margins that are hard to be reached and would have been missed with static SERS imaging alone have been identified, showing great potential for clinical translation and testing. In addition, a similar SERS probe (**Figure 10**A, a) prepared by the same group was used as a molecular imaging agent for liver malignancies 15. In mouse models of hepatocellular carcinoma and histiocytic sarcoma, liver tumors in both cases were readily identifiable with Raman imaging (**Figure 10**A, b-c) after intravenous injection of SERS probes. More importantly, microscopic lesions in the liver and spleen could also be detected by Raman imaging based on the SERS probe, holding promise for improved resection of liver cancer.

A new generation of SERRS probes, which are termed SERRS nanostars, has also been proposed by Kircher and his co-workers. The SERRS nanostars contain a star-shaped gold core, a resonant Raman reporter, and a silica layer (**Figure 10**B, a). In mouse models of various cancers (*e.g.*, pancreatic cancer, breast cancer, prostate cancer, and sarcoma), the as-designed probe enabled the accurate detection both of macroscopic malignant lesions and microscopic disease, without the requirement of a targeting moiety (**Figure 10**B, b-c) 187. Furthermore, thanks to the high sensitivity of the probe, premalignant lesions of pancreatic and prostatic neoplasia have also been successfully imaged. Additionally, this new generation of SERRS probe has also been designed as a dual-modality contrast agent for combined SERS and PA imaging to delineate brain tumor margin 18. Subsequently, similar SERS probes have been functionalized with specific recognition units for cancer detection and imaging 188, 189. In an earlier study, the probe was modified with RGD-peptide for integrin-targeted SERRS molecular imaging of microscopic spread of glioblastoma 188. Then, Kircher*'*s group devised a ratiometric assay that employed FR-targeted SERRS NPs and nontargeted SERRS NPs for imaging of tumor lesions in a murine model of human ovarian adenocarcinoma, demonstrating the robust delineation of tumor deposits with microscopic precision 189. To this end, tumors as small as 370 µm were detected, exhibiting great potential for intraoperative detection of microscopic residual tumors and reducing recurrence rates.

As discussed above, bioimaging of dysregulated cell-surface biomarkers is a promising way to identify the presence of diseases with high sensitivity and specificity. However, since the expression of biomarkers in tumors is heterogeneous, multiplexed molecular imaging of different disease-related biomarkers simultaneously should be addressed. Liu and co-workers have described such strategies that utilized a series of SERS NPs to enable the multiplexed Raman-encoded molecular imaging of freshly excised human tissues for surgical guidance applications 190, 191. A vital component of the described technique is the use of a quantitative ratiometric-imaging method by simultaneously delivering one non-targeted NP flavor to control for the nonspecific behavior of biomarker-targeted NP flavors, thus eliminating the ambiguities due to nonspecific sources of contrast (**Figure 10**C, a). First, Liu and co-workers performed this method for ratiometric imaging of two biomarkers (EGFR, HER2) simultaneously in tumor xenografts with the help of three NP flavors - EGFR-NPs, HER2-NPs, and isotype-NPs (**Figure 10**C, b). Then, fresh human breast tissue specimens from patients were stained with HER2-NPs and isotype-NPs and were subsequently imaged for intraoperative assessment of surgical margins (**Figure 10**C, c) 191. In an extended study, multiplexed imaging of four biomarkers (HER2, ER, EGFR, and CD44) on excised tissues has been achieved with the designed targeted and nontargeted NPs, enabling the rapid visualization of surgical margin surfaces for intraoperative guidance of lumpectomy. Moreover, a first-ever clinical study was performed, in which 57 fresh specimens were imaged to simultaneously quantify the expression of these four biomarkers, accompanying with 89.3% sensitivity and 92.1% specificity 190.

Lately, Li's group has developed new SERS imaging-guided methods for surgical resection of brain tumors 16, 17, 192. They reported the first example of a system that guided brain-tumor resection by sensing acidic tumor microenvironments 192. In this innovative example, a pair of gold nanoprobes was fabricated, including the alkyne-modified probe (Au-AZ) and the azide-modified one (Au-AK). They existed as monodisperse NPs in neutral environments and can enter a brain tumor by crossing the blood-brain barrier (BBB). The tumor acidic environment would trigger their assembly with the activation of SERRS signals, which was used for tumor acidic margin delineation and intraoperatively guiding tumor excision with the assistance of a handheld Raman scanner. A similar approach with a newly designed pH ratiometrically responsive SERS probe (**Figure 10**D, a) was also proposed by Li and co-workers 17. The probe can determine physiological pH with high sensitivity and can delineate the acidic margin of the tumor (**Figure 10**D, b). Due to the positive correlation between tumor acidity and malignancy, acidic margin-guided surgery was implemented in live animal models by intraoperatively evaluating tissue pH/malignancy of the suspicious tissues in tumor-cutting edges.

### Theranostics

Accurate diagnosis and timely effective treatment are crucial to improving the survival rates and life quality of cancer patients. In this regard, theranostic systems that incorporate precise diagnosis and efficient therapeutics in a single platform have been widely investigated 145. The advancement of nanomaterials with unique properties, such as optics, plasmon, electronic, and multifunction, has opened up new possibilities for the development of theranostics nanoplatforms. It should be noted that, recently, SERS-guided theranostic systems based on smartly designed SERS tags or in combination with other imaging techniques for multimodal diagnostics have attracted considerable attention. In this section, the recent progress in the SERS tag-guided theranostic nanoplatforms, including the SERS tag-based multifunctional theranostics and the SERS-guided multimodal therapeutics, will be reviewed.

**Figure 11:**
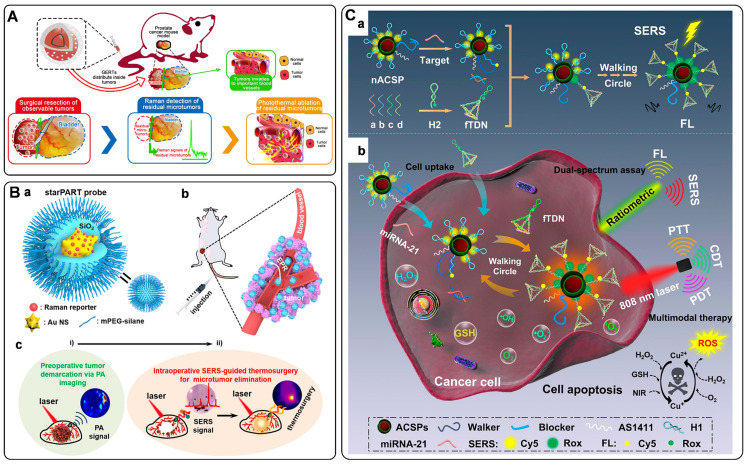
A) Schematic illustration of GERTs for intraoperative detection and eradication of residual microtumors. Adapted with permission from Ref. 19, copyright 2018 American Chemical Society. B) starPART probes for image-guided surgical resection and intraoperative SERS-guided thermosurgical elimination of microtumors. (a) Schematic representation of starPART probes. (b) Targeted delivery of starPART probes via EPR effect in tumor. (c) Schematic illustration of image-guided surgical resection of tumors and real-time intraoperative SERS-guided thermosurgical elimination of residual microtumors. Adapted with permission from Ref. 195, copyright 2021 American Chemical Society. C) Schematic of ratiometric dual-spectrum assay of microRNA and multimodal collaborative tumor therapy. (a) Design of the fTDN-assisted DNA walking nanomachine for simultaneous ratiometric SERS-FL assay of miRNA-21. (b) Illustration of the developed nanodevice for miRNA detection and imaging in live cells and ACSPs-mediated multimodal synergistic therapy. Adapted with permission from Ref. 97, copyright 2021 American Chemical Society.

In view of the excellent plasmonic property of SERS-active substrates, SERS tags are easy to be designed as theranostic agents by coupling SERS bioimaging with PTT 19, 20, 193, 194. For example, GERTs playing dual key roles both for Raman detection and photothermal ablation of residual microtumors have been outlined by Ye's group 19. First, GERTs were injected intravenously, allowing sufficient accumulation inside tumor tissues due to the EPR effects. Then, the bulk tumors were resected, and a 785 nm laser was further applied to elicit the SERS signals of GERTs for imaging the residual tumor lesions. Finally, upon switching an 808 nm laser, the residual tumors were eradicated via PTT (**Figure 11**A). In the orthotopic prostate metastasis tumor model, the GERT-based surgery prevented local recurrence and delivered 100% tumor-free survival. Similar theranostic strategies have also been demonstrated by several other groups. Feng *et al.* synthesized an FA-modified gold nanobipyramids SERS probe for SERS detection and targeted PTT in breast cancer 20. Qiu *et al.* proposed a photodegradable CuS SERS probe for intraoperative residual Raman detection, photothermal ablation, and self-clearance in prostate tumors 193. Lately, Tang and co-workers completed the *in vivo* SERS imaging and PTT of breast tumors using a precise theranostic platform based on a biorthogonal SERS nanotag 194. The SERS nanotags were modified with targeting element of AS1411 and exhibited strong Raman bands in the biologically Raman-silent region, thus can recognize the targeted cancer cells for Raman imaging and PTT with high specificity.

SERS is compatible with other imaging techniques, so that it has been combined with fluorescence 23, PA 195, 196, and MRI 21, 197 to establish theranostic platforms for multimodal imaging, sensing, and therapeutic treatments of tumors. As the typical imaging speed of SERS cannot meet the need for real-time clinical imaging, Kircher and co-workers reported a rational designed fluorescence-Raman bimodal nanoparticles (FRNPs) that combine the specificity of Raman spectroscopy and the speed of fluorescence imaging 23. After selectively accumulation in tumor tissue mouse cancer models, FRNPs enabled real-time fluorescence imaging for tumor resection, followed by Raman-based tumor margin delineation and highly efficient image-guided PTT. Very recently, Wen *et al.* prepared a “three-in-one” theranostic nanoprobe, comprising an AuNSs core, a Raman reporter layer, and a silica outer layer, which draws upon the advantages of PA imaging, SERS detection, and PTT (named as starPART) 195. In *in vivo* experiments, the probe has realized the preoperative PA imaging for surgical resection of tumors, and intraoperative SERS-guided detection for complete PTT of residual microtumors (**Figure 11**B). MRI is a powerful total-body imaging technique that can be incorporated with SERS to produce SERS/MRI dual-mode imaging assays, leading to improved diagnostic accuracy and comprehensive imaging information from the whole body to local tissues. Shen*'*s group outlined a theranostic nanoplatform based on monodispersed Au@PB NPs 21. On the one hand, the iron ions-contained Au@PB NPs exhibit a specific SERS emission in the Raman-silent region, allowing both MRI and accurate zero-background SERS imaging. On the other hand, PB endows remarkably absorption in the NIR region, which can be served as a photosensitizer for PTT and PDT simultaneously. Hence, the Au@PB NPs-based nanoplatform has been acted as an important candidate for SERS/MRI-dependent tumor navigation and *in situ* multimodal therapeutics via PTT/PDT.

Furthermore, SERS-guided multimodal therapeutic strategies have also been presented by other groups 97, 198, 199. For instance, Lee's group reported a hybrid graphene oxide/gold nanoparticle-based cancer-specific nuclei acid conjugate (Au@GO NP-NACs) for multimodal imaging and combined therapeutics of cancer cells. In *in vivo* studies, the as-fabricated nanoprobe showed excellent SERS-mediated tumor detection and imaging, and multimodal synergistic cancer therapy through the use of photothermal, genetic, and chemotherapeutic strategies 198. In another study, Ye and co-workers have developed a cisplatin-loaded GERTs (C-GERTs) nanoprobe for intraoperative Raman detection and elimination of unresectable disseminated advanced ovarian tumors via chemo-photothermal synergistic therapy 199. This C-GERTs nanoprobe can effectively control the spread of disseminated tumors in mice and could serve as a safe and powerful tool for the treatment of advanced ovarian cancers. Very recently, He *et al.* demonstrated an integrated smart nanodevice, composed of Au@Cu_2-x_S@polydopamine nanoparticles (ACSPs) and fuel DNA-conjugated tetrahedral DNA nanostructures (fTDNs), as one stone for many birds (**Figure 11**C) 97. Regarding the analysis, the ACSP probe contained two optical properties: SERS enhancement and fluorescence quenching performance. Employing the ACSP combined with fTDN-assisted DNA walking nanomachines, rapid fluorescence imaging and precise SERS quantitative detection of miRNA in cancer cells was achieved for cancer diagnosis. As a therapeutic application, ACSPs can realize a trimodal synergistic therapy via PTT, PDT, and chemodynamic therapy (CDT). Both *in vivo* and *in vitro* studies verified its biological safety and strong anticancer effect, indicating its promising theranostic applications.

## Conclusion and perspective

This review summarizes the recent progress of SERS tags for biomedical applications. We elaborated three subtopics, including building blocks of SERS tags, biomedical detection and imaging using SERS tags, and SERS tag-guided tumor therapy. The key points lie in designing SERS tags and their biomedical applications. The past decades have witnessed the rapid development of SERS from biological to biomedical applications. Significant progress has been made on improving detection sensitivity and multiplexity. Recent advancements have seen the application of the SERS tags for imaging the pathological tissues, which will be of paramount importance when applied for clinics. In brief, SERS tags possess favorable advantages of high sensitivity, perfect signal specificity, and unparalleled multiplexing capabilities. There are driving forces to exploit these features for significant applications. However, there are still stepping stones that need to reach when employing SERS tags for clinical translation.

Although SERS tags have been successfully used for liquid biopsy, they still have not been widely adopted in POCT. The main bottlenecks are the lack of simple yet high-sensitivity equipment and detection methods. Some portable instruments have been available in recent years. However, their laser power and adjustable ability are somewhat inferior compared with those of large equipment. Apart from developing advanced instruments, future efforts should be made in developing new testing platforms for rapid *in vitro* diagnosis. Integrating SERS tags with advanced POCT devices will be significant for the rapid analysis of biological samples. In addition, multiplex detection capability should also be focused on when employing SERS tags for detecting biomarkers in complex fluids. On the one hand, fabricating non-overlapped SERS tags is an effective strategy for multiplexing detection. This method is adopted by most works mentioned in this review. On the other hand, developing standardized protocols and data processing methods of multiplex analytes are still urgent for accurate quantification. The improvements of the above situations will be critical for future personalized and genomic medicine.

In the case of *in vivo* applications, the biotoxicity and biocompatibility of SERS tags should be first evaluated. Gold and silica are two of the materials approved by the Food and Drug Administration. SERS tags fabricated with these materials have been proved with good biocompatibility and low biotoxicity when employed for cancer diagnosis and therapy. However, the current lack of Raman scanners with a wide field of view and rapid image acquisition speed becomes an obstacle hindering the clinical application of SERS tags. Although a lot of efforts have been made to improve the imaging speed, which mainly concerns the aspects of developing ultra-high bright SERS tags and adopting advanced imaging approaches, the current SERS imaging speed is still slower than fluorescence imaging. Recently, machine learning technologies have been reported to transform the low-resolution image obtained at a rapid acquisition speed into a high-resolution one 200, which may provide a new avenue to speed up the SERS imaging application in the future. In addition, the limited tissue penetration depth also hinders the SERS tags from being employed in full-body or deep tissues. NIR-SERS sensor is promising for SERS tag applied for *in vivo* imaging with high sensitivity and high penetration depth. Further efforts are expected to develop novel SERS substrates and screen more highly sensitive NIR Raman reporters. Last but not least, developing instruments with advanced performance is also an aspect of applying the SERS tags in oncology. With the development of technology, the clinical implementations of SERS tags, such as delineating tumor margins during surgery and visualizing diseased tissues via optical fiber-guided SERS imaging procedures, could find applicability in the near future.

## Figures and Tables

**Table 1:** Typical Raman reporters used for *in vitro* bioanalysis

Raman reporter	Characteristic peak	Application	Reference
4-mercaptobenzoic acid	1586 cm^-1^	detection of oral cancer DNA	38
4-mercaptobenzonitrile	2229 cm^-1^	detection of cancer-associated miRNAs	39
4-(phenylethynyl)aniline	2230 cm^-1^	imaging of biomarkers in cancer cells	40
4-acetamidothiophenol	1073 cm^-1^	detection of alzheimer's disease biomarker	41
4-aminothiophenol	1079 cm^-1^	Detection of miRNA-21	42
4-mercaptophenylacetic acid	1074 cm^-1^	Detection of thrombin	43
1,2-bis(4-pyridyl)ethylene	1612 cm^-1^	determination of thrombin	44
3,3'-diethylthiadicarbocyanine iodide	1133 cm^-1^	Detection of ErbB_2_ biomarker	45
malachite green isothiocyanate	1614 cm^-1^	detection of PSA	46
Rhodamine B isothiocyanate	1643 cm^-1^	Detection of C-reactive protein	47
5,5′-dithio-bis-nitrobenzoic acid	1334 cm^-1^	Detection of telomerase activity	48

## Abbreviations

4-MBA: 4-mercaptobenzoic acid
4-MBN: 4-mercaptobenzonitrile
4-NBT: 4-nitrobenzenethiol
ACSPs: Au@Cu_2-x_S@polydopamine nanoparticles
AgNPs: silver nanoparticles
APTMS: 3-aminopropyltrimethoxy silane
AuNPs: gold nanopartlcles
AuNRs: gold nanorods
AuNSs: gold nanostars
AuTNPs: gold triangle nanoplates
BBB: blood-brain barrier
BDT: 1,4-benzenedithiol
BSA: bovine serum albumin
C3: bladder cancer cell line
CD44V6: CD44 variant isoform 6
CDT: chemodynamic therapy
Ce6: Chlorin e6
CEA: carcinoma embryonic antigen
CHA: catalytic hairpin assembly
CRP: C-reactive protein
CT: computed X-ray tomography
DIPs: dealloyed intra-nanogap particles
DTNB: 5, 5'-dithiobis (2-nitrobenzoic acid)
DTTC: 3, 3'-diethylthiatricarbocyanine iodide
EC: *Escherichia coli*
EpCAM: epithelial cell adhesion molecule
EPR: enhanced permeation and retention
ErbB2: erythroblastic oncogene B2
FA: folic acid
FRNPs: Fluorescence-Raman bimodal nanoparticles
fTDNs: fuel DNA-conjugated tetrahedral DNA nanostructures
GERTs: gap-enhanced Raman tags
GP: poly[4-O-(α-D-glucopyranosyl)-D-glucopyranose]
HBsAg: hepatitis B surface antigen
HCR: hybridization chain reaction
IL-6: interleukin-6
IS: internal standard
LNA: locked nucleic acid
MBs: magnetic beads
MG ITC: malachite green isothiocyanate
MOFs: metal-organic frameworks
MPTMS: 3-mercaptopropyl trimethoxy silane
MRSA: Methicillin-resistant *Staphylococcus aureus*
MUC1: Mucin 1
MRI: magnetic resonance imaging
NIR: near-infrared
PA: photoacoustic
PB: Prussian blue
PCA: principal component analysis
PCR: polymerase chain reaction
PCT: procalcitonin
PDA: polydopamine
PD-L1: programmed cell death receptor ligand 1
PDT: photodynamic therapy
POCT: point-of-care testing
PSA: prostate-specific antigen
PTT: photothermal therapy
RCA: rolling circle amplification
ROS: reactive oxygen species
SA: sialic acid
SAA: serum amyloid A
SBR: signal-to-background ratio
SERRS: surface-enhanced resonance Raman scattering
SERS: surface-enhanced Raman scattering
SH-PEG: mercapto functionalized polyethylene glycol
SLNs: sentinel lymph nodes
SW480: colorectal cancer cell line
TEOS: tetraethyl orthosilicate
UV-vis: ultra violet-visible
ZIF-8: zeolitic imidazolate framework-8
